# A context-dependent bifurcation in the Pointed transcriptional effector network contributes specificity and robustness to retinal cell fate acquisition

**DOI:** 10.1371/journal.pgen.1009216

**Published:** 2020-11-30

**Authors:** Chudong Wu, Jean-François Boisclair Lachance, Michael Z. Ludwig, Ilaria Rebay

**Affiliations:** 1 Committee on Genetics, Genomics and Systems Biology, University of Chicago, Chicago, Illinois, United States of America; 2 Department of Human Genetics, McGill University, Montreal, Quebec, Canada; 3 Department of Ecology and Evolution, University of Chicago, Chicago, Illinois, United States of America; 4 Ben May Department for Cancer Research, University of Chicago, Chicago, Illinois, United States of America; New York University, UNITED STATES

## Abstract

Spatiotemporally precise and robust cell fate transitions, which depend on specific signaling cues, are fundamental to the development of appropriately patterned tissues. The fidelity and precision with which photoreceptor fates are recruited in the Drosophila eye exemplifies these principles. The fly eye consists of a highly ordered array of ~750 ommatidia, each of which contains eight distinct photoreceptors, R1-R8, specified sequentially in a precise spatial pattern. Recruitment of R1-R7 fates requires reiterative receptor tyrosine kinase / mitogen activated protein kinase (MAPK) signaling mediated by the transcriptional effector Pointed (Pnt). However the overall signaling levels experienced by R2-R5 cells are distinct from those experienced by R1, R6 and R7. A relay mechanism between two Pnt isoforms initiated by MAPK activation directs the universal transcriptional response. Here we ask how the generic Pnt response is tailored to these two rounds of photoreceptor fate transitions. We find that during R2-R5 specification PntP2 is coexpressed with a closely related but previously uncharacterized isoform, PntP3. Using CRISPR/Cas9-generated isoform specific null alleles we show that under otherwise wild type conditions, R2-R5 fate specification is robust to loss of either PntP2 or PntP3, and that the two activate *pntP1* redundantly; however under conditions of reduced MAPK activity, both are required. Mechanistically, our data suggest that intrinsic activity differences between PntP2 and PntP3, combined with positive and unexpected negative transcriptional auto- and cross-regulation, buffer first-round fates against conditions of compromised RTK signaling. In contrast, in a mechanism that may be adaptive to the stronger signaling environment used to specify R1, R6 and R7 fates, the Pnt network resets to a simpler topology in which PntP2 uniquely activates *pntP1* and auto-activates its own transcription. We propose that differences in expression patterns, transcriptional activities and regulatory interactions between Pnt isoforms together facilitate context-appropriate cell fate specification in different signaling environments.

## Author summary

Properly patterned tissues require cells transit from a multipotent state to diverse differentiated states in a precise spatiotemporal manner. Meanwhile the programs that direct cell fate adoption must be reliable despite genetic and nongenetic variation. In this study we use the Drosophila photoreceptors as a model system for understanding how specific and robust cell fate transitions are achieved. The specification of seven distinct photoreceptors R1-R7 requires repetitive inductive signaling from the receptor tyrosine kinase (RTK)/ mitogen-activated protein kinase (MAPK). The transcription factor Pointed (Pnt) operates downstream of MAPK to initiate the changes in gene expression appropriate to the particular transition. We asked how the generic MAPK/Pnt signal contributes to different photoreceptor fates. Previous work showed that R1-R7 photoreceptor specification can be subdivided into two rounds that experience different RTK signaling strengths. We find distinct Pnt regulatory networks operate in the two rounds, with the first round network incorporating a novel unstudied Pnt isoform, PntP3. Its inclusion stabilizes developmental transitions when signaling is reduced. We compare and contrast the expression patterns and transactivation potentials of the Pnt isoforms and uncover a web of transcriptional cross-regulation between them. Based on these explorations, we propose that the use of distinct Pnt network topologies provides an adaptive mechanism that permits reliable cell fate transitions under different MAPK signaling environments.

## Introduction

Accurate and reliable transitions from a multipotent state to diverse differentiated states are critical to normal development. A small number of transcription factors acting downstream of an even smaller handful of signal transduction pathways coordinate the gene expression changes that drive cell fate acquisition [1–3]. How these core transcriptional effectors confer both specificity, whereby cells adopt the correct fate in a precise spatiotemporal manner [4–6], and robustness, whereby cells reliably execute the appropriate program despite genetic and nongenetic variations [7,8], to the transitions they oversee remains poorly understood. In this paper we use photoreceptor specification in the developing retina of Drosophila as a model to explore these regulatory mechanisms.

The Drosophila retina is precisely patterned and highly organized. Each of the ~750 ommatidia that comprise the retina contains a core cluster of eight photoreceptors, R1-R8. These neurons are specified in a stereotyped spatiotemporal sequence that is initiated repeatedly as the morphogenetic furrow (MF) travels anteriorly across the epithelial field [9]. Photoreceptor specification occurs in two distinct rounds that are spatially and temporally separated by a single synchronized cell division known as the second mitotic wave (SMW) [10,11]. During the first round, R8 emerges from the morphogenetic furrow (MF)’s wake, followed by the R2/R5 and R3/R4 pairs. Ommatidial assembly then pauses for the SMW, after which the second round of specification recruits photoreceptors R1/R6 and finally R7 to the cluster. Recruitment of non-neuronal support cells to the ommatidia follows immediately, starting with the four lens-secreting cone cells.

Specification of all photoreceptors except R8 requires inductive signaling by the receptor tyrosine kinase (RTK) / Ras / mitogen-activated protein kinases (MAPK) pathway via the transcriptional effector Pointed (Pnt), the Drosophila homologue of the mammalian ETS family activators ETS1 and ETS2 [12,13]. Multipotent retinal progenitors must therefore translate this generic RTK/Pnt signal into specific photoreceptor fates. Numerous studies have focused on combinatorial regulation to integrate the inputs from RTK/Pnt with specific inputs from regionally expressed transcription factors and other signaling pathway effectors. For example, RTK/Pnt, the Spalt transcription factors and Notch signaling collectively specify R4 fates in the first round [14,15] whereas in the second round, RTK/Pnt and Notch signaling integrate with a different transcription factor, Lozenge, to regulate *prospero* transcription and R7 fates [16,17].

Increasing the complexity of these combinatorial codes, RTK signaling inputs are not identical during the two rounds of specification. Fate specification in the first round relies exclusively on signaling initiated by the epidermal growth factor receptor (EGFR), while specification of R1, R6 and R7 second round fates involves a second RTK, Sevenless (Sev) in addition to EGFR [18,19]. Although only R7 fates are lost in a *sev* mutant, the R1 and R6 precursors express Sev, physically contact the Boss ligand-expressing R8 cell, and so are likely to have active Sev signaling [20]. Because both EGFR and Sev use the same Ras/MAPK/Pnt signaling cascade, it has been proposed that cells specified in the second round experience stronger MAPK activation than those in the first round [12,21]. How the Pnt response is tailored to these two different signaling environments has not been explored.

Two Pnt isoforms, PntP1 and PntP2, were identified when the gene was first cloned and have been the focus of subsequent study. The two proteins share the C-terminal ETS DNA binding domain and so are thought to have identical target gene specificity [13,22]. However distinct N-terminal transactivation domains confer both distinct activity and differential regulation by RTK/MAPK signaling [23–25]. Whereas MAPK activation promotes the transcription of *pntP1* [25] to produce the constitutively active PntP1 transcription factor, MAPK regulation of PntP2 occurs post-translationally by direct phosphorylation of a site within its unique N-terminal half [23,24]. Unphosphorylated PntP2 binds target DNA but has very limited transactivation ability; thus phosphorylation by MAPK is required for its full activity [24–27]. Furthermore, expression of a PntP2 mutant in which the phosphoacceptor threonine within the MAPK consensus site was replaced with alanine produces dominant negative effects, consistent with unphosphorylated PntP2 binding and occluding target gene enhancers from appropriate activation [23,24]. Thus the final PntP2 transcriptional output within an individual cell reflects the sum of the low basal, or even the repressive, activity of unphosphorylated PntP2 plus the stronger activity of phosphorylated PntP2, with the availability of active MAPK determining the ratio between the two. The mammalian ETS1 and ETS2 proteins structurally and functionally resemble PntP2 [28].

**Fig 1:**
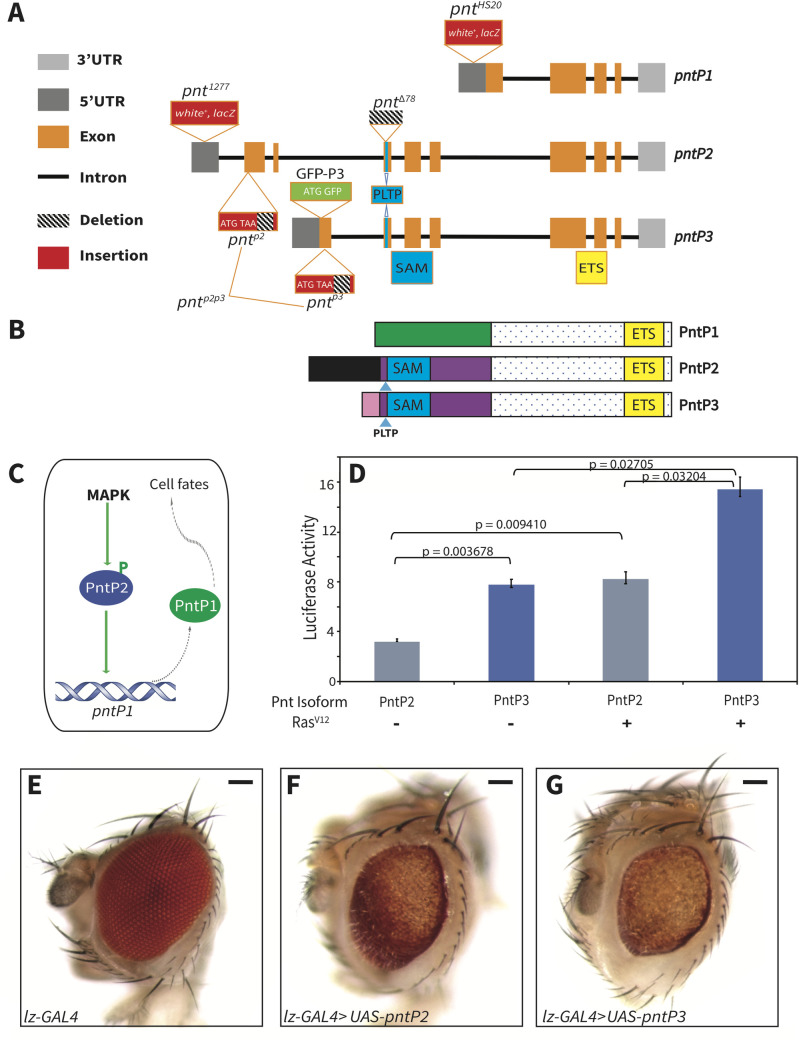
PntP3 is a stronger MAPK-responsive transcriptional activator than PntP2. **(A**) A schematic, not to scale, of the ~55kb *pnt* locus. *pntP1*, *pntP2* and *pntP3* all splice into common 3’ exons encoding the ETS DNA binding domain (yellow box). *pntP2* and *pntP3* share three internal exons encoding the SAM and PLTP MAPK phosphorylation site (blue boxes). Unique N-terminal exons encode isoform-specific sequences. Approximate insertion sites of key P-element-derived alleles are shown: the *white+*, *lacZ* enhancer trap insertions *pnt*^*1277*^ and *pnt*^*HS20*^ respectively report *pntP2* and *pntP1* expression [13]; the excision allele *pnt*^*Δ78*^ disrupts the SAM-encoding exon common to *pntP2* and *pntP3* [24]. Green box labeled ATG-GFP signifies the genomic BAC transgene in which PntP3 was N-terminally GFP tagged. Red boxes labeled ATG-TAA represent the CRISPR-generated null alleles of *pnt*^*p2*^ and *pnt*^*p3*^ that have stop codons immediately after the ATG and exonic deletions; *pnt*^*p2p3*^ carries identical stop codon insertions and deletions. **(B)** A schematic of PntP1, PntP2 and PntP3 proteins highlights their distinct N-termini and common C-termini. The transactivation domains of PntP1 and PntP2 have been mapped to their distinct N terminal regions (green, PntP1; purple, blue and black, PntP2; [28,60]). PntP2 and PntP3 differ only in sequences N-terminal to the MAPK site and SAM (141aa for PntP2. black; 59aa for PntP3, pink). (**C)** A schematic summarizing the sequential activation model: MAPK phosphorylation activates PntP2, phosphorylated PntP2 activates *pntP1* transcription and PntP1 protein drives cell fate specification [25]. **(D)** PntP3 has stronger activity but similar MAPK responsiveness than PntP2 in transcription assays using a reporter with 6 tandem high-affinity ETS sites [24]. For each sample, activity was normalized to reporter alone control. Error bars are S.D. of three independent experiments. P-values were calculated using two tailed pair-wise Student T-tests. **(E-G)** *lz-GAL4*-driven overexpression of *UAS*-*pntP2* (**F)** and *UAS-pntP3* **(G)** disrupts external eye morphology, pigmentation and size relative to driver alone control **(E).** Scale bar: 50 μm.

A previous sequential activation model posited that transient RTK/MAPK signaling activates PntP2, which in turn activates *pntP1* transcription, and that PntP1 then provides a stable, signaling-independent, transcriptional input to the combinatorial codes that initiate the specification of R1-R7 photoreceptor fates (Fig 1C; [25]). However the expression pattern of *PntP2* suggests further complexity, with lower levels in the region of R2-R5 specification and then higher levels in more posterior regions where R1, R6, R7 and cone fates are recruited [25]. These differences parallel the differences in RTK signaling in the two rounds of photoreceptor specification and motivated us to explore how the Pnt response is tuned to these two distinct signaling environments.

In this study, we uncover distinct Pnt regulatory networks for the two rounds of specification that are distinguished by the inclusion/exclusion of another evolutionary conserved but previously unstudied Pnt isoform, PntP3. As predicted by its protein structure, PntP3 functions as a MAPK-responsive transcriptional activator, but with intrinsically higher activity than PntP2. In contrast to R1, R6 and R7 specification where PntP2 is uniquely expressed and required, we find that PntP3 and PntP2 are coexpressed and under wild type conditions function redundantly during specification of first round R2-R5 photoreceptors. However under conditions of compromised signaling, the individual activities of PntP3 and PntP2 are essential to the robustness of these fate transitions. Mechanistically, we uncover distinct auto- and cross-regulatory transcriptional interactions between PntP2 and PntP3 during the two rounds of photoreceptor specification that likely optimize context-specific output, with the most striking a shift from PntP2 auto-repression during specification of first round fates to auto-activation during specification of second round fates. We conclude that a combination of functional redundancy, different transactivation potential and the reset of transcriptional regulatory interactions between Pnt isoforms adapts the transcriptional response to different RTK signaling environments.

## Results

### PntP3 is a MAPK-responsive transcriptional activator whose expression overlaps that of PntP2 in R2-R5 photoreceptors

Although the field has focused on the two isoforms identified when the *pnt* gene was first cloned [13,22,24,25,29], the BDGP cDNA project together with subsequent high-throughput mRNA sequencing has revealed additional transcripts (S1A Fig; [30–32]). First, there is a second *pntP1* isoform (*pnt-E* in G-Browse) identical to “classic” *pntP1* (*pnt-C*) except for a longer 3’UTR and an encoded product with an extra two amino acids owing to the use of an alternate splice donor site at the 3’ end of the first coding exon. Second, there is a transcript identified as *pnt-D* that is closely related to but distinct from *pntP2* (*pnt-B*); we refer to this novel isoform as *pntP3*.

*pntP3* is distinguished from *pntP2* by its unique transcription start site, 5’UTR and N-terminal coding exons, but then like *pntP2*, it splices into the exons encoding the sterile alpha motif (SAM) and adjacent MAPK consensus site, and the ETS domain (Fig 1A and 1B). To our knowledge there have not been any explicit studies of *pntP3*. However, just like *pntP1* and *pntP2*, *pntP3* was detected by RNA-Seq profiling in most developmental stages [31], suggesting it might contribute to the transcriptional response downstream of RTK signaling. Further, both PntP2 and PntP3 are conserved across *Drosophila* species from *D*. *melanogaster* to *D*. *virilis* (S1B and S1C Fig). Conservation across millions of years suggests strong evolutionary pressure for keeping both PntP2 and PntP3, implying essential functions.

Given the protein-level similarity, we began by asking whether PntP3, like PntP2, functions as a transcriptional activator positively regulated by MAPK phosphorylation. In transcriptional reporter assays in transiently transfected S2 cells, PntP3 was about two-fold more active than PntP2 in the absence of MAPK stimulation; MAPK stimulation induced a further ~two-fold activity increase for both isoforms (Fig 1D). When overexpressed in the developing eye, PntP3 also showed greater activity than PntP2, producing stronger disruptions of adult eye morphology with all Gal4 drivers tested (Figs 1E, 1F, 1G and S2). These phenotypes were associated with ectopic induction of photoreceptor fates in 3^rd^ instar discs (S2 Fig), consistent with previous studies of Pointed overexpression [25]. As predicted by their relative activities in S2 cells, ectopic expression of neuronal markers was more striking with *pntP3* overexpression than with *pntP2* (S2A–S2F Fig). The expression level and subcellular localization to the nucleus were indistinguishable between the two isoforms (S2G–S2I Fig), indicating differential transcriptional activity most likely underlies the phenotypic differences.

In addition to the activity differences between PntP2 versus PntP3 that we attribute to the unique sequences at the N-terminal ends of their transactivation domains (Figs 1B, S1B and S1C), the use of separate 5’ regulatory regions suggested that expression pattern differences might also distinguish their developmental roles. To explore this, we compared their endogenous expression in late 3^rd^ instar eye discs where *pnt* function is essential for photoreceptor specification and has been well studied [13,23,33]. We relied on the *pntP2*-specific enhancer trap allele *pnt*^*1277*^ [13,25] to report PntP2 expression. To visualize PntP3 expression, we inserted an N-terminal GFP tag in a genomic BAC that contains the entire *pnt* locus (GFP-P3, Fig 1A) and that we had previously shown to be fully functional [34]. The *GFP-PntP3* transgene fully complemented the lethality of *pnt*^*Δ88*^*/Df(pnt)* animals, a background null for all three isoforms, producing phenotypically wild type, fertile adults.

**Fig 2:**
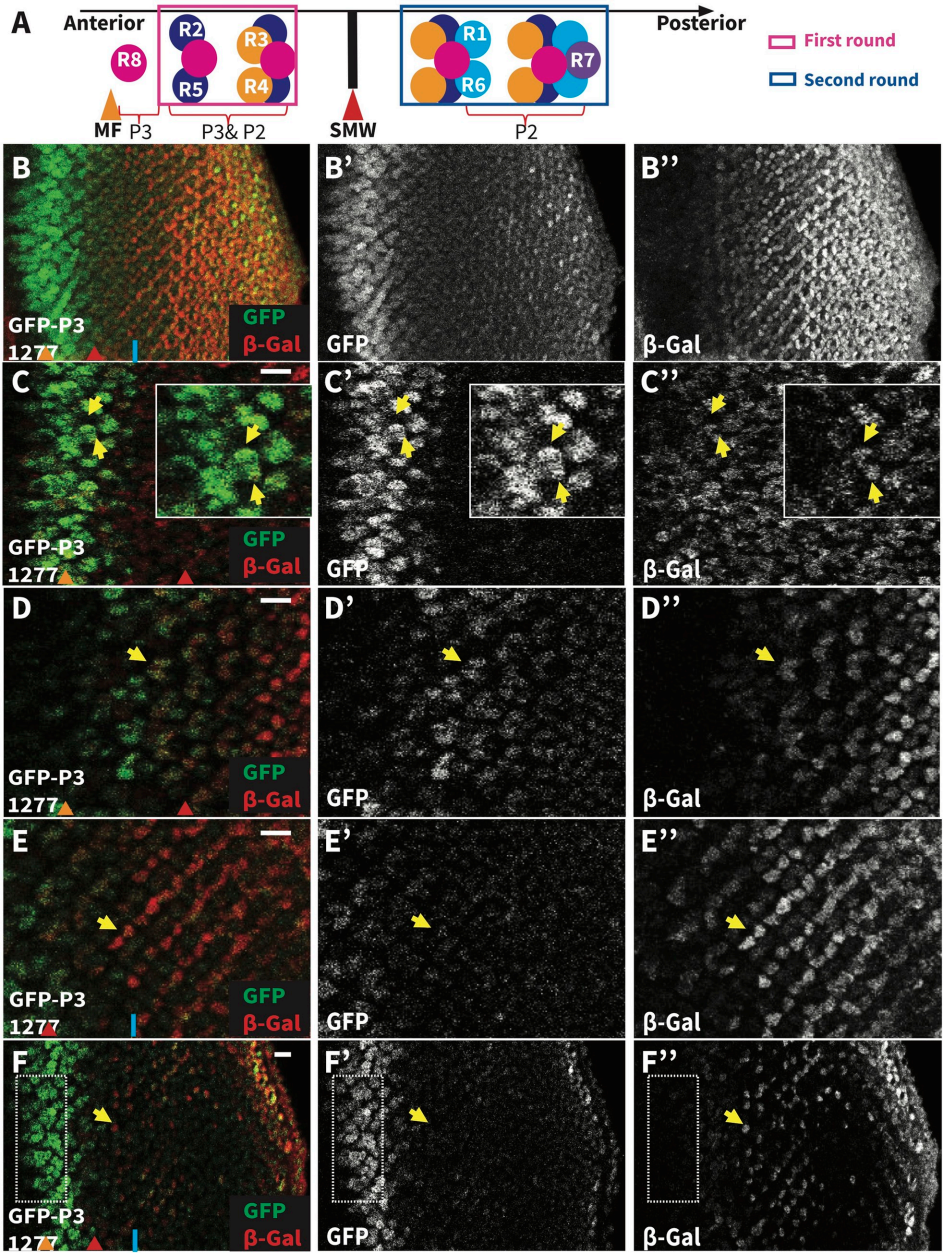
PntP3 and PntP2 show overlapping and complementary expression patterns. **(A)** A schematic summarizing the sequential specification of photoreceptor fates and the expression patterns of PntP3 and PntP2. R8 cells are specified first near the MF (orange arrowhead) and express PntP3. R2/R5 and R3/R4 pairs are specified next (first round fates) and express both PntP3 and PntP2. After the SMW (red arrowhead), R1/R6 and R7 are specified (second round fates) and express PntP2. **(B-F**) Representative 3rd instar eye imaginal discs, oriented anterior left, comparing the pattern of *pntP2* transcription (red), as reported by *pnt*^*1277*^, and PntP3 protein (green), as reported by a GFP-PntP3 genomic BAC transgene. (B) Maximum projection highlighting the complementary pattern of highest PntP3 in the MF region and highest *pntP2* posterior to the SMW (blue line). (B’, B”) Single channel images show that *pntP2* transcription starts anterior to the SMW in cells where GFP-PntP3 is expressed. (C-F) Single optical slices of the disc in (B) at different apical/basal planes. (C, D) Coexpression was detected in R2/R5 pairs (C, yellow arrows, insets show zoomed view) and in R3/R4 pairs (D, yellow arrows). (E-F) *pntP2* but not PntP3 was detected in R1/R6 pairs (E, yellow arrows) and in R7 (F, yellow arrows). PntP3 but not *pntP2* was detected in basal progenitors at the MF (F, boxed region). Scale bar: 10 μm.

Analysis of 3^rd^ instar eye discs dissected from animals carrying both *GFP-PntP3* and the *pntP2*-specific enhancer trap allele revealed both distinct and overlapping patterns of expression (Fig 2). Lower magnification projections emphasized the complementary aspects of the two patterns, with GFP-PntP3 expression strongest in and immediately posterior to the MF and β-galactosidase (β-gal) reporting strongest *pntP2* expression in the posterior half of the eye field (Fig 2B). As the stereotyped differentiation sequence of ommatidial assembly means every cell can be unambiguously identified by its position and morphology [10,11,35,36], higher magnification views at different optical planes enabled cell type specific comparison of the two patterns (Fig 2C, 2D, 2E and 2F). Coexpression was detected in R2/R5 and R3/R4 photoreceptor pairs, in basal progenitors at the MF and in cone cells (Figs 2C, 2D and S3A). Complementary expression was detected posterior to the second mitotic wave (SMW) in photoreceptors R1, R6 and R7 where *pntP2* was high and GFP-PntP3 low, and in apically localized nuclei at the MF, including R8, where GFP-PntP3 was high and *pntP2* low (Fig 2E and 2F). The combined differences and similarities in cell type specific expression patterns raised the possibility of both distinct and overlapping functional requirements for PntP2 and PntP3 in the two rounds of photoreceptor specification (Fig 2A).

### Redundant and non-redundant requirements for PntP2 and PntP3 during two distinct rounds of photoreceptor specification

Prior studies of *pnt* function during retinal development concluded that *pntP1* and *pntP2* are required non-redundantly to specify photoreceptors R1-R7 [24,25]. However the *pntP2* allele used in the studies, *pnt*^*Δ78*^ [24], was generated by imprecise excision of a P-element inserted into the first SAM-encoding exon, and so also disrupts *pntP3* (Fig 1A). This means that *pnt*^*Δ78*^ phenotypes, in the eye loss of R1-R7, reflect the combined loss of *pntP2* and *pntP3*.

To reveal the individual requirements for the two isoforms we generated *pnt*^*p2*^ and *pnt*^*p3*^ specific mutants using CRISPR/Cas9 genome editing (Fig 1A). To confirm the effectiveness of the molecular strategy, we also engineered a *pnt*^*p2p3*^ double mutant allele. As reported for *pnt*^*Δ78*^ [24,37], homozygous *pnt*^*p2p3*^ adults were never recovered, indicating that the combined function of the two isoforms is essential for viability. In contrast, homozygous *pnt*^*p3*^ animals were fully viable while homozygous *pnt*^*p2*^ animals occasionally survived (scoring 2058 3^rd^ instar progeny from a cross between *pnt*^*p2*^*/TM6B* parents found only 35 homozygous *pnt*^*p2*^ animals, a 1.7% survival rate). The differences in survival of the isoform specific mutants suggested both redundant and non-redundant requirements for PntP2 and PntP3 during development, with PntP2 playing the major role and PntP3 a more auxiliary one.

**Fig 3:**
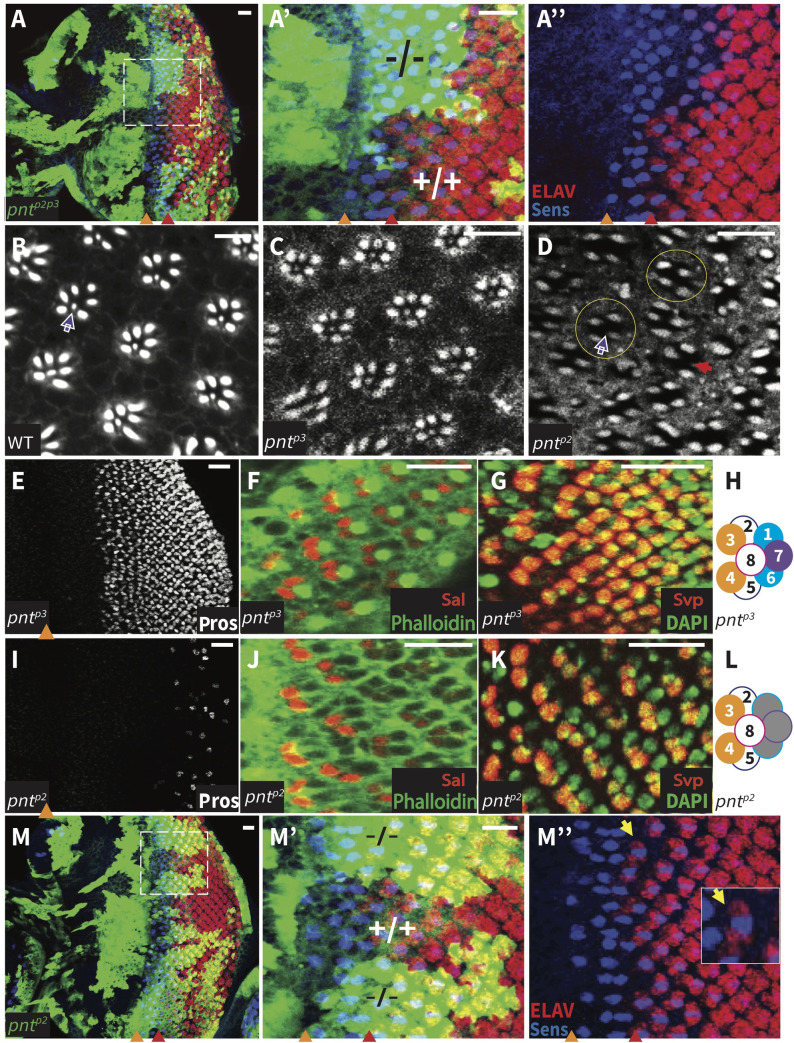
Redundant and unique requirements for PntP2 and PntP3 in photoreceptor specification. **(A)** Representative 3^rd^ instar eye disc of genotype *eyFLP/+;act-Gal4*,*UAS-GFP;FRT82B*,*pnt*^*p2p3*^*/tub-Gal80*,*FRT82B*, oriented anterior left. Elav (red) marks all photoreceptors and anti-Sens (blue) marks R8. Homozygous *pnt*^*p2p3*^ mutant clones, positively marked by GFP (green), lack all photoreceptors except R8. A’ and A” show zoomed views of boxed region in A. MF, orange arrowhead. SMW, red arrowhead. Scale bar: 10 μm. **(B-D)** Adult retinas stained with phalloidin to mark the rhabdomeres. (B, C) Both wild type and *pnt*^*p3*^ mutants have regularly arrayed rows of ommatidia; white arrow points to the small R7 rhabdomere at the center of the outer trapezoid formed by the larger R1-R6 rhabdomeres. (D) All ommatidia of homozygous *pnt*^*p2*^ mutants lack R7, most also lack two outer rhabdomeres (yellow circles), and some show even greater loss (red arrow). Scale bars: 5 μm. **(E-L)** Representative 3rd instar eye discs, oriented anterior left. R7 and cone cells are marked by Pros (white), R3/R4 pairs are marked by Sal (red) or Svp (red), and R1/R6 pairs are marked by Svp (red). (E-G) *pnt*^*p3*^ mutants appear wild type. (I-K) *pnt*^*p2*^ mutants lack R7 and most cone cells (I), have normal R3/R4 specification (J, K), and lack R1/R6 (K). (H, L) Schematic summaries of photoreceptor specification patterns in *pnt*^*p3*^ and *pnt*^*p2*^ mutants. Scale bars: 10 μm. **(M)** Representative 3^rd^ instar eye disc of same genotype as in (A). Homozygous *pnt*^*p2*^ clones, positively marked by GFP (green), show normal R8/R2/R5 specification as marked by Elav and Sens. K’ and K” show zoomed views of boxed region in K; yellow arrow points to a newly specified R2/R5 pair. Scale bar: 10 μm.

Focusing on photoreceptor specification, homozygous *pnt*^*p2p3*^ clones were missing all photoreceptors except R8 (Fig 3A), consistent with published analysis of *pnt*^*Δ78*^ [24,25]. We reasoned that if the function of both PntP2 and PntP3 is required for photoreceptor specification, then neither single mutant should recapitulate the double mutant phenotype. If so, the requirement for PntP3 should manifest in the first round fates where it is strongly expressed but not in second round fates where its levels are low (Figs 2A, 2B and S3B). Alternatively, if PntP3 does not contribute activity essential to photoreceptor specification, then the *pnt*^*p2*^ and *pnt*^*p2p3*^ mutants should show identical loss of R1-R7 phenotypes.

To test these predictions, we first assessed photoreceptor loss in adult eyes, using F-actin to highlight the number and spatial arrangement of the rhabdomeres. In a wild type ommatidium, the larger rhabdomeres of R1-R6 are arrayed in a trapezoidal-shaped ring around the smaller R7 rhabdomere (Fig 3B). Whereas ommatidia of homozygous *pnt*^*p3*^ adults had the full complement of photoreceptors (Fig 3C), those of the rare homozygous *pnt*^*p2*^ escapers did not (Fig 3D). Loss of R7 was fully penetrant (100%, n = 204, white arrow), with most ommatidia missing two additional photoreceptors (73%, n = 204, yellow circles), and some showing even greater loss (red arrow). The more modest photoreceptor loss seen in *pnt*^*p2*^ single mutants relative to *pnt*^*p2p3*^ double mutants indicates a functional requirement for PntP3.

Examination of 3^rd^ instar discs confirmed that loss of *pnt*^*p2*^ resulted in loss of only the cell fates recruited during the second round of specification. First, only a few Pros-positive cells remained in the posterior of discs from homozygous *pnt*^*p2*^ animals; a similar posterior scattering of Cut-positive cells suggested a complete failure to specify R7 photoreceptors and most cone cells (Figs 3I, S4A and S4B). Second, normal expression of Sal and reduction of Svp expression to only two, rather than four cells per ommatidia, indicated correct specification of photoreceptors R3/R4 and a failure to specify R1/R6 (Fig 3J, 3K and 3L). Third, and confirming no other consistent photoreceptor specification defects, examination of Sens and Elav patterns in *pnt*^*p2*^ mosaic discs indicated normal recruitment of photoreceptors R8/R2/R5 (Fig 3M and 3L). Thus the complete loss of R1-R7 fates that occurs in *pnt*^*p2p3*^ double mutant ommatidia (Fig 3A) reflects the combined loss of redundant inputs to R2-R5 first found fates plus the PntP2-specific input to R1, R6 and R7 second round fates.

### PntP2 and PntP3 provide robustness through redundant activation of pntP1 transcription

A central tenet of the current model of *pnt* function during photoreceptor specification is that PntP2 activates *pntP1* transcription (Fig 1C; [25]). Given the partial genetic redundancy between PntP2 and PntP3, we asked whether PntP3 also contributes to this activation.

**Fig 4:**
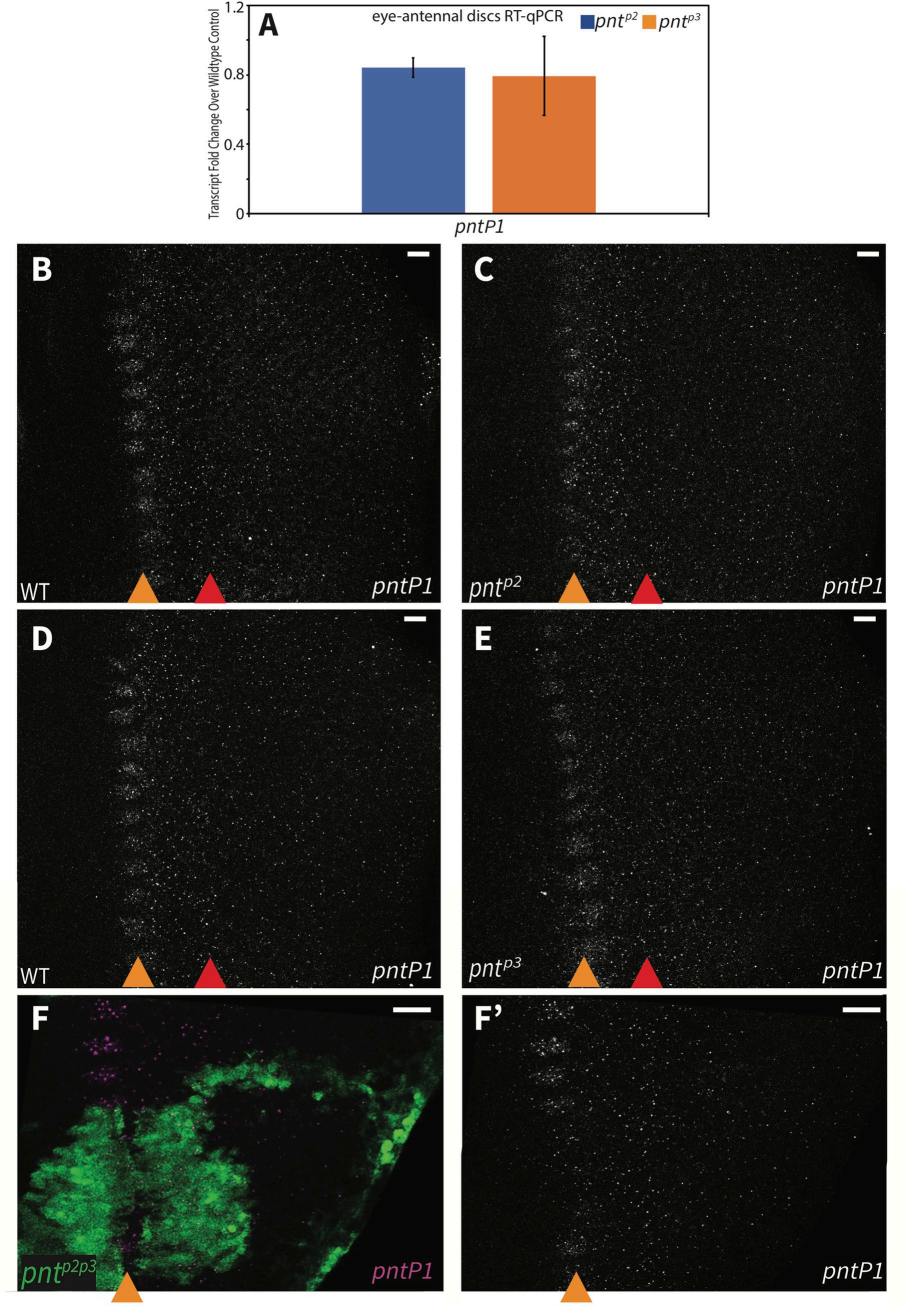
PntP2 and PntP3 redundantly activate *pntP1* transcription. **(A)** RT-qPCR comparison of *pntP1* transcript levels in wild type versus *pnt*^*p2*^ (blue bars) and *pnt*^*p3*^ (orange bars) 3^rd^ instar eye-antennal discs. No significant changes were detected. Error bars represent S.D. of three independent experiments. Significance was calculated via pair-wise Student T-tests between the mutant sample and the control gene. **(B-F)** *pntP1* FISH in 3^rd^ instar eye imaginal discs, oriented anterior left, with MF marked by orange arrowhead and SMW marked by red arrowhead. (B-E) Maximum projections. (F) partial projections. Scale bars: 5 μm. (**B, C**) *pntP1* transcripts patterns were comparable between wild type (B) and *pnt*^*p2*^ (C). **(D, E)** *pntP1* transcript patterns were comparable between wild type (D) and *pnt*^*p3*^ (E). **(F)** Homozygous *pnt*^*p2p3*^ mutant clones, positively marked with GFP (green), show reduced *pntP1* at the MF. Consistent results were obtained from analyzing 12 clones from 9 discs across 3 independent experiments.

To start, we used reverse transcription quantitative polymerase chain reaction (RT-qPCR) to measure *pntP1* transcript levels in *pnt*^*p2*^ and *pnt*^*p3*^ mutant tissues. In both mutants, decreases in *pntP1* transcripts were measured in eye discs across three independent biological replicates, although the changes were not statistically significant (p = 0.1; Fig 4A and S1 Table). Repeating the analysis in wing discs also failed to detect significant changes (S5A Fig). This suggests either redundancy between PntP2 and PntP3 with respect to activating *pntP1* transcription, inadequate sensitivity in the RT-qPCR assay, or that PntP2 and PntP3 are not the primary activators of *pntP1*.

We were concerned that by grinding up whole tissue we were destroying spatial information and therefore missing locally significant changes in *pntP1* levels. Also, the animals lacking both PntP2 and PntP3 do not survive to 3^rd^ instar, precluding RT-qPCR analysis of the double mutant. Thus we turned to fluorescence in situ hybridization (FISH) to ask whether the two isoforms work redundantly to active *pntP1* expression. FISH probes targeting the *pntP1* isoform-specific exons revealed an expression pattern consistent with that of the *pntP1* enhancer trap allele [13,25]. Specifically, we detected peak *pntP1* transcription in a periodic pattern at the MF, lower levels of expression in the zone between the MF and SMW region and then lowest levels posterior to the SMW (Figs 4B, 4D and S5B). In pair-wise comparisons of wild type vs. *pnt*^*p2*^ and wild type vs. *pnt*^*p3*^, no changes in *pntP1* transcription were noted (Fig 4B, 4C, 4D and 4E), consistent with the RT-qPCR results. However in *pnt*^*p2p3*^ mutant clones, *pntP1* expression at the MF was strongly reduced (Fig 4F). We conclude that PntP2 and PntP3 redundantly activate *pntP1*.

### Context specific auto- and cross-regulation of *pntP2* transcription

Having established the functional redundancy of PntP2 and PntP3 with respect to induction of *pntP1*, we next investigated how the system tunes these two parallel inputs. In particular we wondered whether cross-regulatory feedback might coordinate and optimize PntP2/PntP3 expression levels, and ultimately their activity. To test this, we used RT-qPCR to measure changes in *pntP2* and *pntP3* transcript levels in eye imaginal discs dissected from *pnt*^*p2*^ and *pnt*^*p3*^ homozygous mutant 3^rd^ instar larvae.

**Fig 5:**
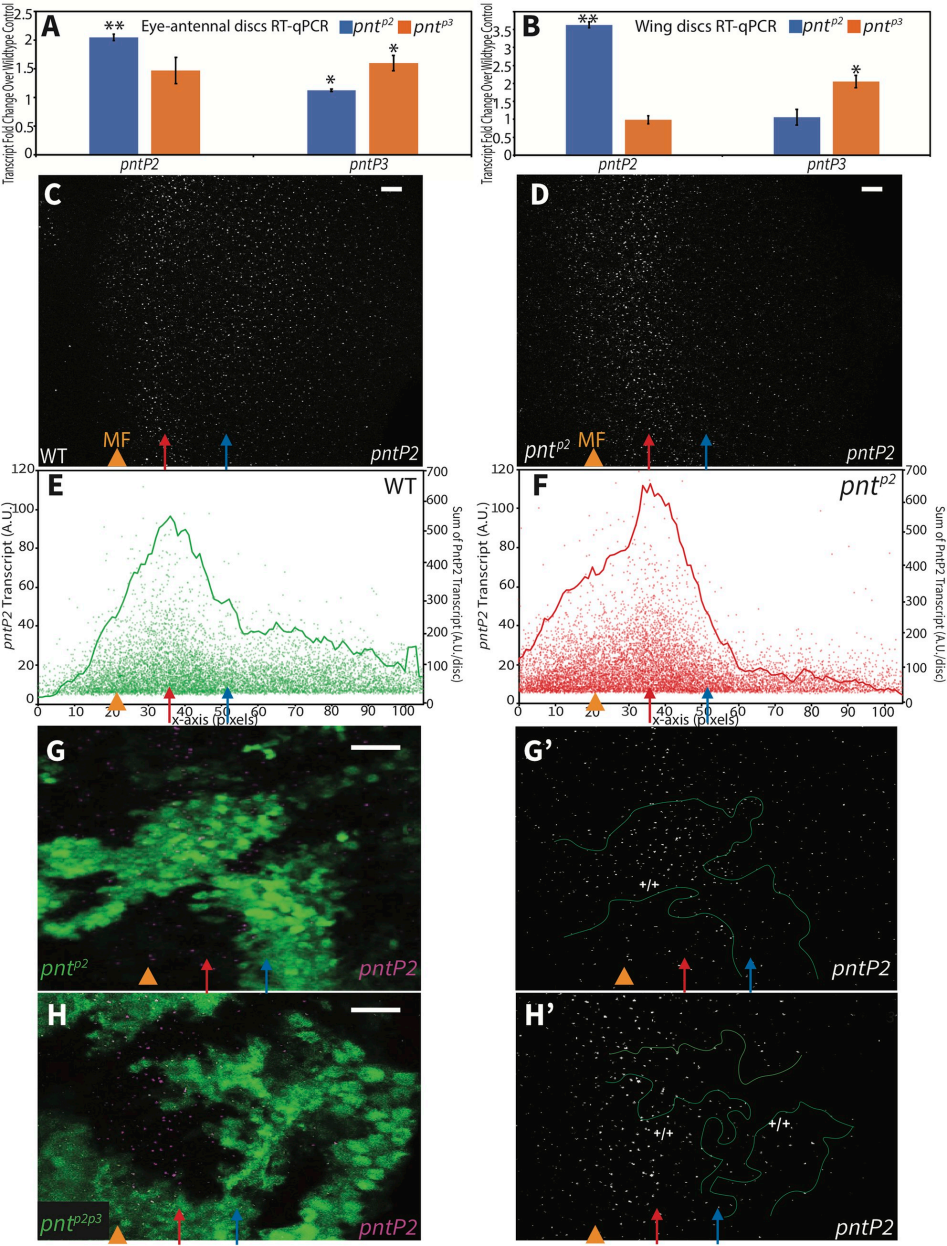
Distinct context-specific interactions regulate *pntP2* transcription across the eye field. **(A, B)** RT-qPCR comparison of *pntP2* and *pntP3* transcript levels in wild type versus *pnt*^*p2*^ (blue bars) and *pnt*^*p3*^ (orange bars) 3^rd^ instar eye-antennal (A) and wing (B) discs. Significant increases were detected. Error bars represent S.D. of three independent experiments. Significance was calculated via pair-wise Student T-tests between the mutant sample and the control. **, p < 0.01; *, p< 0.05. **(C, D)** Maximum projection images of *pntP2* FISH in representative wild type and *pnt*^*p2*^ 3^rd^ instar eye imaginal discs, oriented anterior left. Orange arrowheads mark the MF, red arrows mark the peak of *pntP2* expression and blue arrows mark the start of lower expression in the posterior half of the disc; the three can be mapped to correspondingly colored marks in Fig 2A based on the pixel distances. In *pnt*^*p2*^ discs (D) relative to wild type (C), an increased and broader peak of *pntP2* transcripts was detected in and immediately posterior to the MF while a decrease was seen in the posterior half of the disc. Scale bar: 5 μm. **(E, F)** Quantification of *pntP2* FISH in 6 wildtype (E) and 6 *pnt*^*p2*^ mutant (F) discs from maximum projections. In wild type, *pntP2* levels begin to rise anterior to the MF, peak and decrease to a steady state. In *pnt*^*p2*^ discs, *pntP2* levels were higher than normal in the anterior half (left of blue arrow) but lower in the posterior (right of blue arrow). Each dot plots the product of the fluorescent intensity and the size of an individual *pntP2* FISH focus, representing the relative amount of *pntP2* transcript (y-axis on the left) The line connects the moving average of the sum of all foci within one-pixel windows along the x-axis (y-axis on the right). **(G)** Homozygous *pnt*^*p2*^ clones in a 3^rd^ instar eye disc, positively marked with GFP (green). Clone boundary is circled with green line (G’). *pntP2* levels in the mutant clones appeared higher in the anterior region but decreased in the posterior relative to adjacent wild type tissue. Examination of 8 clones in 7 discs from 3 independent experiments showed consistent changes. Images are partial projections. Scale bar: 5 μm. **(H)** *pntP2* FISH in homozygous *pnt*^*p2p3*^ clones, positively marked with GFP (green). *pntP2* levels in anterior mutant clones were indistinguishable from wild type but appeared decreased in more posterior clones. Examination of 9 clones in 6 discs from 2 independent experiments showed consistent changes. Images are partial projections. Scale bar: 5 μm.

Two findings emerged. Most striking, and unexpectedly, the experiment uncovered negative auto-regulation for both isoforms (Fig 5A). Thus, *pntP2* transcript levels were significantly increased in *pnt*^*p2*^ mutant tissue (p < 0.01) and *pntP3* transcripts were significantly increased in *pnt*^*p3*^ mutant tissue (p < 0.05). Given the surprising nature of this result, we repeated the experiment using wing imaginal discs, and again found significant increases in transcript levels in the respective mutant (Fig 5B). Thus both isoforms negatively regulate their own transcription, either directly or indirectly.

Second, evidence of cross-regulation emerged from the eye disc experiments (Fig 5A), with a modest, but reproducible and significant 12% average increase in *pntP3* transcript levels measured in *pnt*^*p2*^ mutant tissue (p < 0.05; S1 Table). In the converse experiment, the increases in *pntP2* transcript levels measured in *pnt*^*p3*^ mutant tissue were more variable across the three biological replicates (S1 Table), resulting in a statistically insignificant 47% average increase (p = 0.18). Thus the possibility of bidirectional inhibitory cross-regulation remains an open question. Cross-regulatory interactions were not detected in the wing disc (Fig 5B).

The coexpression of PntP2 and PntP3 in the anterior half of the disc where first round photoreceptor fates are specified predicted that the regulatory interactions uncovered by RT-qPCR were occurring in this context. We therefore turned to FISH to corroborate the negative auto-regulation and to assess further the possibility of cross-regulatory interactions. We found that *pntP2* transcription initiated at the MF, peaked in the region of the second mitotic wave (SMW), and then continued at a more moderate level across the posterior half of the disc (Fig 5C, 5D and 5E). This pattern was consistent with that reported by the enhancer trap *pnt*^*1277*^ although the prolonged perdurance of beta-galactosidase likely over-reports *pntP2* levels in the posterior (Fig 2). Unfortunately our FISH protocol was not able to detect *pntP3*, presumably because its specific exon is too short for adequate numbers of probes (see Materials and Methods).

We next compared *pntP2* transcript levels in wildtype versus *pnt*^*p2*^ null mutant retinal tissue. In both whole mutant eye discs (Fig 5C, 5D, 5E and 5F) and in null mutant clones (Fig 5G), increased *pntP2* transcription was evident in the MF and in the adjacent region where *pntP2* levels normally peak (Figs 5C and S6). We also noticed a change not predicted by the RT-qPCR analysis, namely a decrease in *pntP2* transcripts in the posterior of the disc (Figs 5C, 5D, 5E and 5F and S6). This suggests *pntP2* transcription is regulated differently in anterior versus posterior regions of the developing eye field.

Because PntP3 expression is strongest anteriorly (Fig 2), we wondered whether the increase in *pntP2* transcripts detected in *pnt*^*p2*^ mutant tissue reflected cross-regulatory activation by PntP3. To test this we examined *pntP2* transcript levels in *pnt*^*p2p3*^ double mutant clones (Fig 5H). No increase was detected at the MF or in the adjacent region of peak expression. In more posterior *pnt*^*p2p3*^ mutant clones, *pntP2* transcript levels were lower than in adjacent wild type clones, exactly as seen in *pnt*^*p2*^ single mutant clones (Fig 5G). Thus in anterior regions where PntP3 expression is strong, loss of PntP2 results in a PntP3-dependent increase in *pntP2* transcription whereas in posterior regions where PntP3 expression is normally low, loss of PntP2 results in a PntP3-independent reduction in *pntP2* transcription. This suggests that the regulatory relationships between Pnt isoforms established at the MF are reset after the SMW.

### PntP2 and PntP3 buffer developmental transitions against compromised RTK signaling

**Fig 6:**
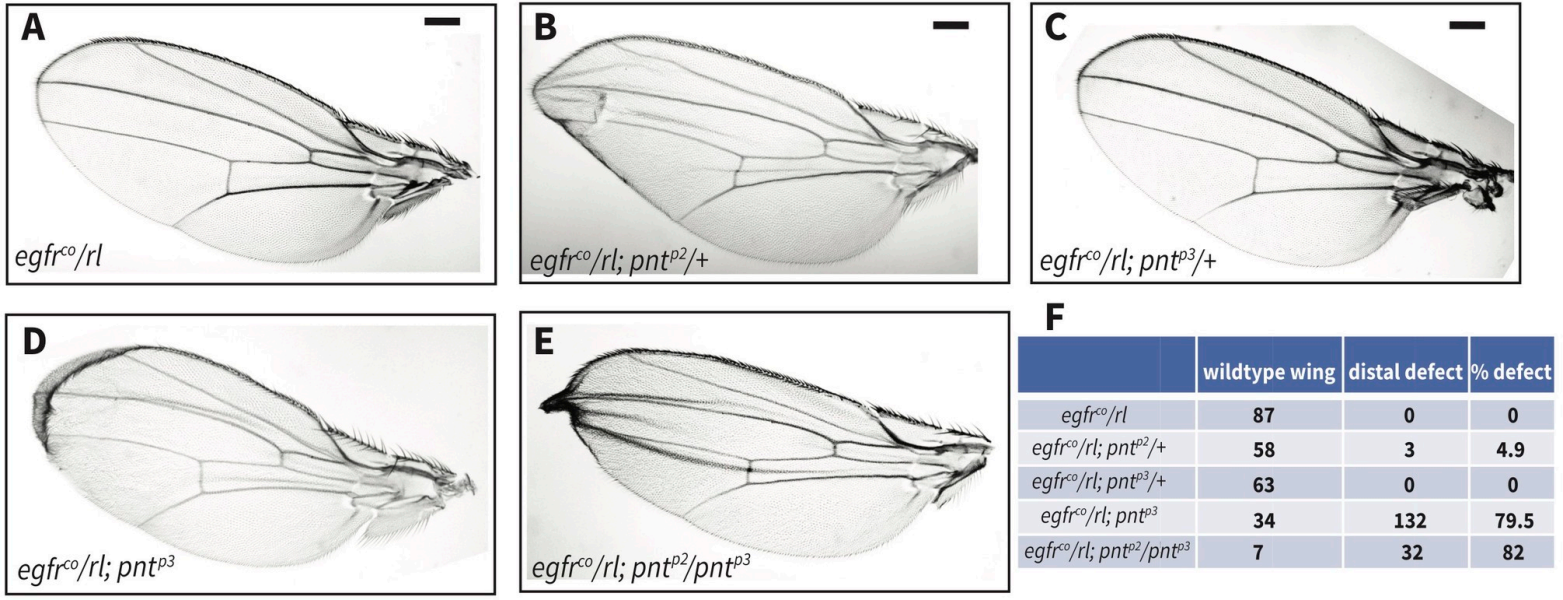
PntP2 and PntP3 buffer against the reduction of MAPK activation during wing patterning. **(A-E)** Representative adult wings, oriented anterior up and distal left, showing the effects of reduced *pnt*^*p2*^ and *pnt*^*p3*^ dose in a sensitized *egfr*^*co*^ */rl* background. (A) *egfr*^*co*^*/rl* trans-heterozygotes appeared wild type. (B) Loss of one copy of *pnt*^*p2*^ produced occasional distal margin defects. (C) Loss of one copy of *pnt*^*p3*^ did not disrupt patterning. (D) Loss of both copies of *pnt*^*p3*^ resulted in penetrant distal margin defects. (E) Simultaneous reduction in dose of *pnt*^*p2*^ and *pnt*^*p3*^ synergistically increased wing margin defects. **(F)** Quantification of wings as either wild type or with margin defects for each genotype in (A-E). Scale bar: 0.1 mm.

Functional redundancy can provide robustness not only toward loss-of-function mutations in each gene, but also toward variation in the signaling environment in which the gene products function; such variation can result from either genetic or environmental stress. Given that Pnt mediates RTK/MAPK signaling, we asked whether the inclusion of PntP3 in the network protects development from being perturbed when RTK signaling is compromised. We first reduced pathway activity by removing one copy each of *egfr* and of the MAPK encoding gene *rolled (rl)*. We grew the animals either at constant optimal temperature (25°C) or subjected them to repeated 18°C to 31°C temperature shifts. Indicating the remaining activity was sufficient to support normal development, adult *egfr/rl* heterozygotes appeared fully wild type (Figs 6A and S7). In the absence of temperature stress, reducing the dose of either *pntp2* or *pntp3* did not produce retinal defects, but in the wing, another context in which PntP2 has been implicated in EGFR-mediated regulation of patterning [38], defects were noted (Fig 6B and 6C). Most striking, removal of either both copies of *pnt*^*p3*^ or one copy each of *pnt*^*p2*^ and *pnt*^*p3*^ in the *egfr/rl* background resulted in an 80% penetrant disruption in pattern (Fig 6D, 6E and 6F). This suggests that PntP3 confers robustness in situations when RTK/Pnt signaling levels fall below a certain threshold. When temperature stress was added, *egfr/rl; pnt*^*p3*^ animals died as pharate adults, but again without significant photoreceptor loss (S7 Fig). However in retinas dissected from pharate *egfr/rl; pnt*^*p2*^/*pnt*^*p3*^ quadruple heterozygotes, 12% of ommatidia were missing photoreceptors (S7 Fig). Thus the strong genetic synergy noted between *pntP2* and *pntP3* during wing patterning (Fig 6F) is also important for retinal development.

**Fig 7:**
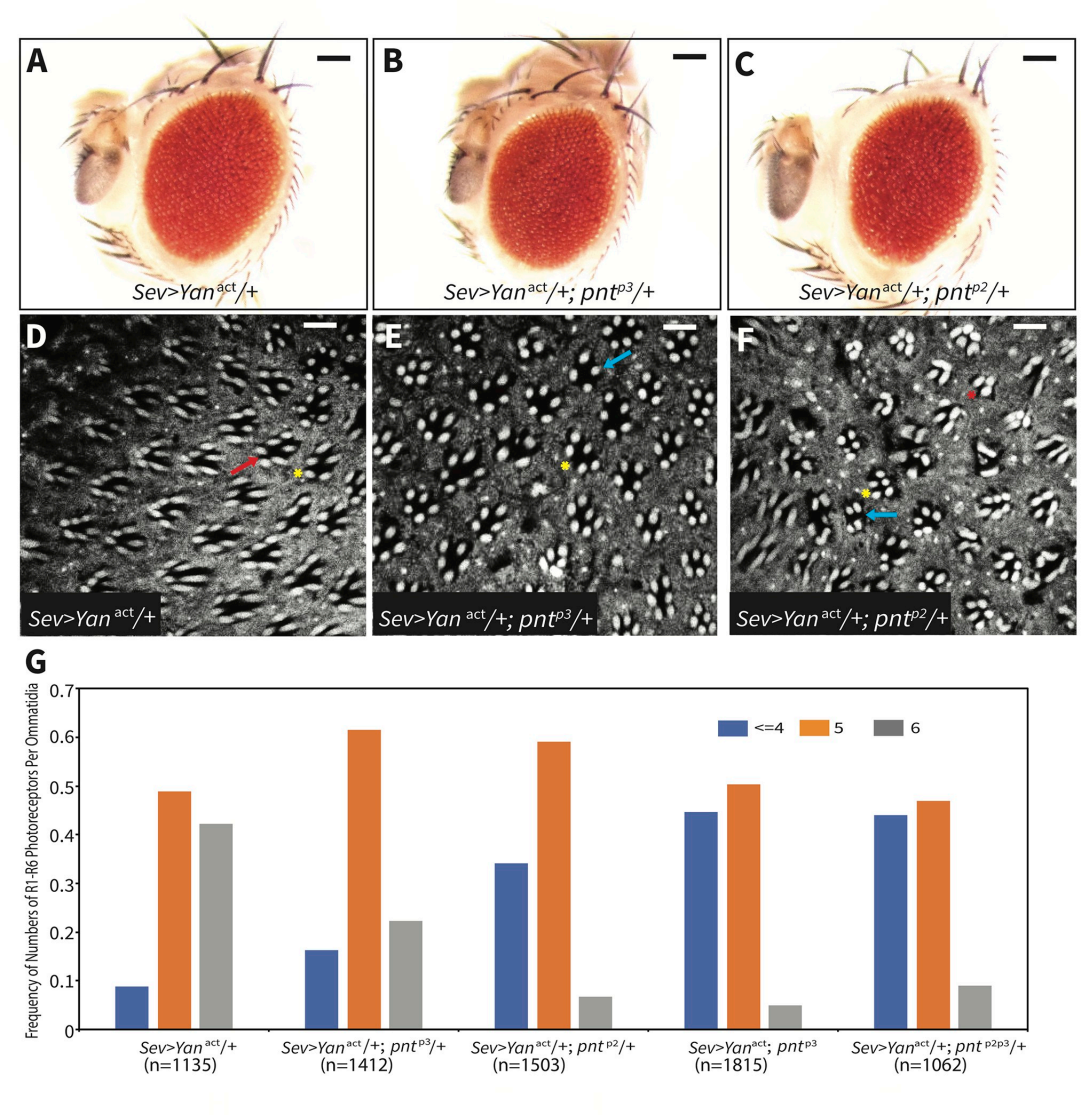
PntP2 and PntP3 stabilize R3/R4 fate transitions against compromised RTK signaling. **(A-F)** Heterozygosity for either *pnt*^*p3*^ or *pnt*^*p2*^ dominantly enhances the *Sev-Yan*^*act*^ induced disruption in external eye morphology and photoreceptor loss. (A-C) Representative adult eyes of indicated genotype. Scale bar: 50 μm. (D-F) Phalloidin staining of representative adult eyes of indicated genotype. Red arrow points to the ommatidium lacking the R7 rhabdomere, yellow stars indicate ommatidia with five outer rhabdomeres, blue arrows point to ommatidia with four outer rhabdomeres and red star indicates the ommatidium with three outer rhabdomeres. Scale bar: 5 μm. **(G)** Quantification of photoreceptor loss in phalloidin-stained adult eyes expressed as the frequency of ommatidia with 6, 5, or < = 4 remaining outer rhabdomeres. n represents the number of ommatidia scored for each genotype. In the *Sev-Yan*^*act*^ controls, ommatidia with 5 or 6 outer rhabdomeres were present at a roughly 1:1 ratio and less than 10% of ommatidia had fewer than 4. Heterozygosity for either *pnt*^*p3*^ or *pnt*^*p2*^ shifted this distribution: for both, the majority of ommatidia (~60%) had 5 outer rhabdomeres. However reduction in *pnt*^*p2*^ caused a larger increase in fraction of ommatidia with 4 or fewer outer rhabdomeres than did reduction in *pnt*^*p3*^. When both copies of *pnt*^*p3*^ were removed, ommatidia with < = 4 or 5 outer rhabdomeres were present at a roughly 1:1 ratio and less than 10% of ommatidia had 6 rhabdomeres. A similar enhancement pattern was scored in animals heterozygous for the double mutant *pnt*^*p2p3*^.

Encouraged by these findings, we turned to a more extreme sensitized background to test further the robustness hypothesis in the retina. Specifically we crossed the *pnt*^*p2*^ and *pnt*^*p3*^ alleles to flies carrying a *Sev-Yan*^*ACT*^ transgene, a genetic background in which constitutive activity of the RTK antagonist Yan blocks specification of the photoreceptor fates in which it is expressed [39,40]. The *sev* regulatory sequences drive expression mainly in the R3/ R4 pair, in R7 photoreceptors and in cone cells [41,42]. We took advantage of the dose-sensitivity of *Sev-Yan*^*ACT*^ transgenes [39,40] and selected a line that causes fully penetrant loss of R7 but only partial loss of R3/R4 photoreceptors; as a result, overall disruption to the adult eye pattern is modest (Fig 7A, 7D and 7G; [40]). Zooming in and quantifying the number of rhabdomeres in over a thousand ommatidia showed that about ~50% of *Sev-Yan*^*ACT*^ ommatidia were missing either an R3 or R4 rhabdomere (Fig 7D, yellow star and Fig 7G, orange bar); and so in contrast to those only missing R7 but retaining the normal complement of 6 large rhabdomeres representing the R1-R6 photoreceptors (Fig 7D, red arrow and Fig 7G, grey bar), these only had 5 rhabdomeres. Thus, in this genetic background, RTK signaling is just barely sufficient to support R3/R4 specification. Because PntP2 and PntP3 are co-expressed and functionally redundant in normal R3/R4 fate specification (Figs 2 and 3), this provided an ideal context to assess whether this redundancy provides robustness to compromised signaling.

Removal of one copy of either *pntP2* or *pntP3* dominantly enhanced the *Sev-Yan*^*ACT*^ rough eye phenotype, producing visibly stronger disruptions in the adult eye pattern (Fig 7A, 7B and 7C). Quantification of rhabdomere numbers across thousands of individual ommatidia supported this qualitative impression and revealed a shift toward more penetrant photoreceptor loss (Fig 7E, 7F and 7G). Thus introducing heterozygosity for either *pnt*^*p2*^ or *pnt*^*p3*^ reduced the frequency of ommatidia with the full complement of R1-R6 rhabdomeres to only ~10–20% (Fig 7G, grey bars) and increased the frequency of R3/R4 loss (Fig 7G. orange and blue bars). Loss of either both copies of *pnt*^*p3*^ or one copy each of *pnt*^*p2*^ and *pnt*^*p3*^ enhanced even further, with quantification showing similar patterns of increased photoreceptor loss (Figs 7G, S8A and S8B). In these enhanced backgrounds, ommatidia with fewer than four rhabdomeres were occasionally found (Fig 7F, red star); this could reflect the additional loss of R1 or R6 cells, where Sev drives expression at much lower levels [20,41] or a later consequence of cone cell loss [39] on overall photoreceptor survival. Overall these results reveal non-redundant contributions of both PntP2 and PntP3 to R3/R4 photoreceptor specification under conditions of reduced RTK signaling.

## Discussion

In this study we explore the contributions of a previously uncharacterized Pointed isoform, PntP3, to the transcriptional effector network that directs developmental transitions downstream of receptor tyrosine kinase signaling. We show that PntP3, like PntP2, functions as a MAPK responsive transcription factor, but that despite their molecular and functional similarities, PntP3 and PntP2 have distinct expression patterns, transcriptional activities and mutant phenotypes. Together our results suggest that essential regulatory responsibilities previously attributed solely to PntP2, are actually distributed between PntP2 and PntP3, and that depending on context, the two work redundantly, uniquely or synergistically. We speculate that a network of auto- and cross-regulatory interactions between the isoforms fine-tunes Pnt transcriptional output to confer specificity and robustness to the developmental transitions it directs.

Our investigation of the PntP3 isoform has uncovered a context-dependent bifurcation in the transcriptional effector network that transduces RTK/MAPK signaling. In doing so, it has also corrected an erroneous assumption regarding the role of the closely related PntP2 isoform. Prior to our study, the accepted model was that MAPK phosphorylation of PntP2, followed by PntP2p-mediated induction of *pntP1* transcription, provided the essential activating input for RTK-dependent transitions (Fig 1C; [25]). As exemplified by studies in the eye, the genetic cornerstone of this model was that null alleles of either *pntP2* or *pntP1* produce identical phenotypes of failing to specify R1-R7 fates [24,25,33]. However the allele *pnt*^*Δ78*^ [24], previously misinterpreted as a *pntP2-*specific null, actually disrupts the exon common to *pntP2* and *pntP3*. Thus the failure to specify R1-R7 fates reflects the compound loss of PntP2 and PntP3. We note that an earlier study using hypomorphic truly *pntP2*-specific alleles concluded correctly that there is an “absolute requirement for *pntP2* function in R1, R6 and R7” but did not detect the requirement in R2-R5 [23].

**Fig 8:**
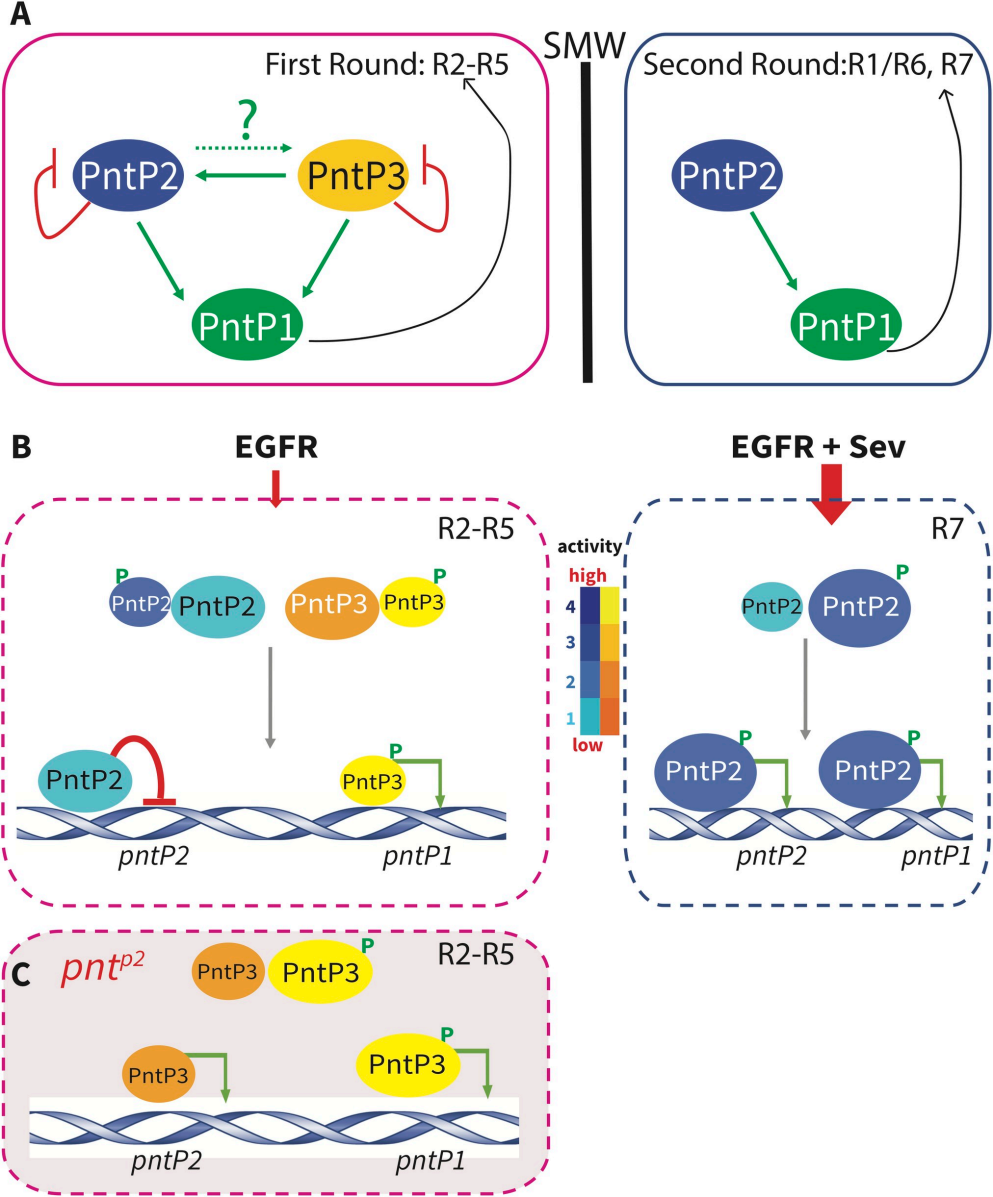
Model: Context-specific topology and function of the Pnt network. **(A**) A schematic summary of the Pnt network. During first-round specification, both PntP2 and PntP3 auto-repress their transcription, PntP3 activates *pntP2*, and PntP2 and PntP3 redundantly activate *pntP1*. During the second-round, PntP2 auto-activates its own transcription and activates *pntP1*; PntP3 does not contribute. **(B)** Proposed Pnt network functions in the different signaling environments of first and second round specifications. The color scheme illustrates the range of transactivation activity: for PntP3, yellow indicates high activity and orange low; for PntP2 dark blue indicates high activity and light blue low. As depicted for PntP2, the low transactivation potential of the unphosphorylated forms may allow the proteins to operate as transcriptional repressors. Different sized ovals depict relative abundance of the phosphorylated versus unphosphorylated forms. See Discussion for details.

A schematic summarizing the combined contributions of PntP3 and PntP2 to photoreceptor fate specification is presented in Fig 8 as a framework for considering some of the mechanistic implications of our work. To recap briefly the key phenotypes and regulatory interactions on which the model is based, our study revealed redundant functional requirements for PntP2 and PntP3 in specifying the first round fates R2/R5/R3/R4; thus either single mutant recruits wild type 5-cell ommatidial clusters, while only in the double mutant are R2-R5 fates lost. Molecularly, PntP2 and PntP3 redundantly activate *pntP1* transcription (Fig 8A) with significant reduction in *pntP1* levels detected only in the double mutant. In contrast, only PntP2 is required during the second round of photoreceptor specification and so eyes from isoform-specific *pnt*^*p2*^ null mutants lack R1, R6, R7 fates whereas *pnt*^*p3*^ mutant ommatidia are wild type. Because *pntP1* transcript levels posterior to the SMW are already quite low in wild type discs, our FISH experiments were unable to detect the presumed reduction in *pntP1* in *pnt*^*p2*^ mutant discs.

As a general developmental strategy, the redundant use of PntP2 and PntP3 may provide an effective buffer against genetic perturbations that reduce RTK signaling. Using R2-R5 photoreceptor specification as a specific example, the presence of redundant MAPK effectors in the early stages of ommatidial assembly may maximize overall robustness by minimizing early “mistakes” that would derail the entire process. Supporting this idea, we found that in a genetically sensitized background with reduced MAPK signaling output in R3, R4 precursors [39,40], loss or reduction in dose of either *pntP2* or *pntP3*, which in otherwise wild type discs did not compromise patterning, now resulted in loss of these cell fates. Analogous results were obtained in the wing, and when temperature stress was added on top of genetic stress, animals lacking PntP3 failed to eclose. Thus redundant use of PntP2 and PntP3 can confer developmental robustness.

Just as inadequate signaling compromises developmental transitions, so will excessive, oncogenic-levels of pathway activation. For example, genetic perturbations that enhance RTK pathway output, such as increased Pnt expression or activity, severely disrupt ommatidial assembly and wing patterning [29,43,44]. Therefore to prevent redundant use of PntP2 and PntP3 from overactivating transcriptional programs, the Pnt output needs to be fine-tuned.

The negative auto-regulation of both *pntP2* and *pntP3* transcript levels uncovered in our study may serve this purpose (Fig 8A). Although Pnt is well-established as a transcriptional activator, a handful of studies have implicated Pnt in negative regulation of gene expression [45–48]. The underlying molecular mechanisms are still under investigation, but based on our prior work showing extensive Pnt chromatin occupancy across the *pnt* locus [48], we favor a mechanism in which direct auto-repression keeps PntP2 and PntP3 levels in check. However an indirect mechanism involving Pnt-mediated transcriptional activation of a repressive factor is equally plausible. If direct auto-regulation is used, the ability of Pnt to recruit and co-occupy enhancers with the ETS family repressor Yan and the corepressor Groucho uncovered in a recent study [48] could provide the repressive mechanism.

Counteracting the negative auto-regulation at *pntP2* and *pntP3*, we also uncovered positive transcriptional cross-regulation whereby PntP3 can activate *pntP2*. Thus in *pnt*^*p2p3*^ double mutant clones, the increase in *pntP2* transcript levels that occurs in *pnt*^*p2*^ single mutants was no longer observed. Again, we favor the simplest model of direct activation of *pntP2* by PntP3 (Fig 8A), but cannot rule out more complicated indirect regulatory relays. Whether the converse cross-regulation of *pntP3* transcription by PntP2 occurs, and whether PntP2 and/or PntP3 positively auto-regulate their transcription anterior to the SMW remains to be assessed. The decrease in *pntP2* transcript levels measured posterior to the SMW in *pnt*^*p2*^ mutant tissue argues that positive auto-regulation is possible, making it plausible that such regulation could also fine-tune PntP2/PntP3 levels and output during specification of first round fates.

How specific PntP2:PntP3 ratios influence the acquisition of different photoreceptor cell fates will be in an interesting focus for future work. Numerous studies have shown that regulatory networks can either amplify or suppress both the intrinsic noise (i.e. the randomness associated with transcription and translation) and extrinsic noise (i.e. the fluctuations in cellular processes or environment) of protein levels to influence cell fate decisions [49–51]. Very speculatively, perhaps the network of auto-repressive and cross-activating interactions between PntP2 and PntP3 also tunes the cell-to-cell variation in Pnt isoform or Pnt target gene expression, thereby influencing the response to inductive signaling.

Another intriguing feature of the network of transcriptional interactions uncovered in our study is that corresponding to the switch from redundancy between PntP2 and PntP3 to the uniqueness of PntP2, the balance of PntP2 autoregulation shifts from repression during first round fate specification to activation during the second round (Fig 8A). Fig 8B offers speculation on how the distinct RTK signaling environments anterior vs. posterior to the SMW, combined with intrinsic differences in PntP2 vs. PntP3 activity, could produce this shift. Briefly, we propose that the level of MAPK activity determines the ratios between the unphosphorylated forms of PntP2 and PntP3 and the phosphorylated forms, PntP2p and PntP3p, and that these ratios in turn dictate specific transcriptional output.

For example, during photoreceptor specification, R2-R5 first round fates rely exclusively on EGFR signaling while the R1, R6, R7 photoreceptors specified during the second-round experience additional RTK signaling through Sevenless (Sev); studies focused on R7 specification have highlighted the requirement for both EGFR and Sev [21,52,53]. Using the same Ras/MAPK/Pnt pathway, EGFR and Sev-initiated signals can be considered interchangeable [12,54], with lower pathway activity required in the first round and higher activity needed in the second [20,21]. Because both PntP2 and PntP3 are direct MAPK substrates whose transactivation potential is increased by phosphorylation, their combined transcriptional output will be sensitive to the abundance of activated MAPK. Under conditions of lower signaling and when both isoforms are co-expressed, as occurs anterior to the SMW, competition for the limited pool of activated MAPK will lead to domination by the unphosphorylated, less active forms. The presence of PntP3, whose unphosphorylated form has equivalent activity to PntP2p, and whose phosphorylated form has twice the activity of PntP2p (Fig 1D), may be important to make sure pathway output remains above a certain threshold in situations with lower levels of signaling. Although at first glance this might predict that the system would not tolerate loss of PntP3, because loss of PntP3 also reduces MAPK substrate competition, this would shift the distribution of PntP2 protein toward the phosphorylated more active form, thereby ensuring a robust transcriptional response.

How might these relationships manifest at the level of target gene enhancers? Given that PntP2 and PntP3 have the same ETS DNA binding domain and are identical except for the sequences N-terminal to the SAM, we expect they recognize the same DNA binding sites. Thus in the simplest scenario in which the phosphorylated and unphosphorylated forms of both PntP2 and PntP3 compete equally for enhancer occupancy, situations in which the unphosphorylated forms predominate would prevent excess activation of target genes. Much greater regulatory complexity is possible if modest enhancer-specific preferences between PntP2 and PntP3 and between the phosphorylated and unphosphorylated forms bias the competition. We suggest such biased competition will be essential to achieving limited activation, or even repression, of target genes such as *pntP2*, while allowing strong induction of others, such as *pntP1*, in the same cell. Based on a large-scale interactome study that reported closely related isoform pairs often have distinct protein-protein interaction patterns [55], it is possible that association with distinct cofactors also contributes to Pnt isoform enhancer occupancy bias.

The substrate competition-based model also readily explains the transcriptional shifts that may occur in the individual *pnt*^*p2*^ and *pnt*^*p3*^ mutants (Fig 8C). If one removes either PntP2 or PntP3, then overall competition for activated MAPK is eased, resulting in domination by the phosphorylated form of the remaining protein to boost transcriptional output. This would derepress targets like *pntP2*, as detected in our experiments, while activation of targets like *pntP1* would continue at physiologically functional levels. This same scenario plays out in an even stronger form in the wild type disc during specification of second round photoreceptor fates, where the combination of only PntP2 plus twice the RTK pathway input would result in phosphorylation of an even greater proportion of total PntP2 protein (Fig 8B). Because PntP2 appears to have intrinsically weaker transactivation potential than PntP3, ensuring full phosphorylation in situations where it is the sole MAPK effector may be critical to activating the transcriptional program.

Besides its role in the specification of first round photoreceptor fates, the expression of PntP3 suggests it might also contribute to the patterning of R8 and the cone cells. For R8, although its specification does not require EGFR/Pnt activation, EGFR signaling is essential for the proper spacing between R8 cells [56–59]. As GFP-PntP3 and *pntP1* both express at high levels at the MF, they may have redundant functions in R8 spacing. For cone cells, previous work has suggested that as with first round photoreceptor fates, EGFR is the sole RTK involved in their specification [12]. Our finding that GFP-PntP3 is coexpressed with *pntP2* in cone cells, combined with the partial loss of Cut-positive cells that we noted in *pnt*^*p2*^ mutant discs, suggests PntP3 and PntP2 may together ensure robust specification of cone cell fates. Further investigation of the function and regulation of Pnt isoforms in a broad range of developmental contexts will be an interesting direction for further studies.

Our study adds to the growing appreciation of the enormous regulatory potential available to developing tissues through the combinatorial expression and use of different protein isoforms, and also offers insights beyond the *Drosophila* arena. The human homologs ETS1 and ETS2, although encoded by separate genes, show intriguing structural and functional parallels to the *Drosophila* PntP2 and PntP3 isoforms [28,60]. For example, ETS1 and ETS2 have distinct sequences at the N-terminal end of the conserved transactivation domain, similar MAPK responsiveness, and overlapping but not identical functions and expression patterns [28,61]. Given these striking parallels, continued exploration of the molecular mechanisms underlying Pnt-mediated transcriptional responses may provide new insight into signaling robustness and specificity in mammalian systems.

## Materials and methods

### Drosophila strains

From the Bloomington *Drosophila* Stock Center: *Lz-Gal4*, *pnt*^*Δ88*^, *pnt*^*1277*^, *FRT82b*, *egfr*^*co*^. Additional strains: *ro-GAL4* [62], *Sev-yan*^*ACT*^ [40], *rl*^*S135*^ [43], *ey-FLP; act-Gal4*, *UAS-GFP/CyO; FRT82b*, *tub-GAL80/TM6B* (a gift of Wei Du, University of Chicago, IL, USA), *UAS-flag-pntP2*, *UAS-flag-pntP3*, GFP-PntP3, *pnt*^*p2*^, *pnt*^*p3*^, *pnt*^*p2p3*^ (this work). Flies were cultured at 25°C on standard cornmeal-molasses-agar medium unless otherwise indicated.

*UAS-flag-pntP2* and *UAS-flag-pntP3* flies were made by amplifying the cDNA sequences with oligos P2-forward- 5’GTTGGTACCGAATTGGCGATTTGTAAAACAGATCTGTCTGC3’ or P3-forward- 5’GTTGGTACCACCAATGAGTGGATCGATTGGAATGACAGT3’, and reverse- 5’CGCTCTAGACTAATCCACATCTTTTTTCTCAATCTTAAGATCATACTTGGC3’, subcloning into pUASt-FLAG-attB and integrating into the φC31 86FB landing site [63].

GFP-PntP3 flies were made by recombineering the BAC (CH321-39L2, 3R:23,266,463..23,357,204 [+]) [64] to introduce a monomeric GFP tag at the N-terminus immediately after the ATG. BAC transgenes were inserted into the VK37 site on the second chromosome.

To compare the transcriptional activity of PntP2 and PntP3 in the developing eye, we crossed *lz-Gal4* or *ro-Gal4* to *UAS-pntP2* or *UAS-pntP3*. To compare the expression of *pntP2* and *pntP3*, we crossed *pnt*^*1277*^ to GFP-PntP3. Mosaic eye clones were made by crossing *ey-FLP; act-Gal4*, *UAS-GFP/CyO; FRT82B*, *tub-GAL80/TM6B* to *pnt*^*p2*^, *FRT82B*/*TM6B* or *pnt*^*p2p3*^, *FRT82B*/*TM6B*. To assess loss of *pntP2* and/or *pntP3* in the background of *Sev-yan*^*ACT*^, we crossed *Sev-yan*^*ACT*^/*CyO* to *pnt*^*p2*^*/TM6B* or *pnt*^*p2p3*^*/TM6B* or *pnt*^*p3*^. To assess loss of *pntP2* and/or *pntP3* in the background of reduced EGFR signaling, we crossed *egfr*^*co*^*/CAG* (*CAG* = *CyO*,*Act>GFP*) or *egfr*^*co*^*/CAG*; *pnt*^*p2*^*/TM6B* to *rl*^*SR135*^*/CAG* or *rl* ^*SR135*^*/CAG; pnt*^*p3*^*/TM6B*.

### Temperature stress

Following the experiment published by Li et al 2009 [65], flies of the relevant genotypes were crossed at 25°C and transferred to fresh bottles daily. Cultures were maintained at 25°C until larvae reached early third instar, shifted to 31°C for 16–24 hr, and then subjected to seven to ten rounds of temperature cycles. Each round consisted of a shift to 18°C for 2 hours followed by 31°C for 1.5–2 hours. After the final round, the larvae were returned to 25°C and retinas were dissected from eclosed or pharate adults. Bottles were incubated in air-circulating incubators for each temperature step.

### Transcription assays

2.25 x 10^6^ of *Drosophila* S2 cells plated in 12-well plates in 1.5 mL of Schneider’s medium (Sigma) were transfected in duplicate with a mixture of dimethyl- dioctadecyl-ammonium bromide (DDAB) (Sigma) containing 100 ng of 6X-ETS luciferase reporter construct, 100 ng of PntP2/pMTHA or 100 ng of PntP3/pMTHA, 20 ng of actin >Renilla luciferase, and if applicable, 5 ng of Ras^V12^/pMT. After 48 hr, cells were lysed in 170 uL transcription assay lysis buffer (100 mM potassium phosphate, 0.5% NP-40, pH7.8), and incubated on ice for 30 min. Luciferase measurements were made using an Autolumat Plus LB 953, using luciferase buffer (10 mM Mg acetate, 100 mM tris acetate, 1 mM EDTA, pH 7.8) with 4.5 mM ATP (Fisher) and 77 uM D-luciferin (Pierce), and Renilla buffer (25 mM sodium pyrophosphate, 10 mM Na acetate, 15 mM EDTA, 500 mM Na_2_SO_4_500 mM NaCl, pH 5.0) with 4 mM coelenterazine (Promega). Empty vector was used to standardize the amount of DNA transfected across conditions. Luciferase measurements were made in technical triplicates (50 uL per sample) for each biological replicate, and the ratio of Firefly RLU to Renilla RLU was taken as transcriptional activity, and then all measurements were normalized to reporter alone with empty vector. For statistics, significance was calculated via pair-wise Student T-tests between the samples indicated. **, p < 0.01; *, p< 0.05.

### Immunohistochemistry and microscopy

For antibody staining, third instar eye-antennal imaginal discs were dissected in S2 cell medium, fixed for 10 min in 4% PFA with 0.1% Triton X-100, washed 3X in PBT (1X PBS, 0.1% Triton), blocked in PNT (1X PBS, 0.1% Triton, 1% normal goat serum), stained with primary antibodies in PNT overnight at 4° C, washed 3X in PBT, and stained with secondary antibodies in PNT overnight at 4° C. Adult tissues were treated in the same manner, except that halved heads were pre-fixed for 20 min prior to dissecting the retinas, and then post-fixed for 10 min. For endogenous GFP, third instar eye-antennal imaginal discs were fixed in 4% PFA without Triton for 30 minutes, incubated with DAPI for 10 min and mounted immediately. Imaging was performed with a Zeiss LSM 880 confocal microscope, using 0.8 to 1.0 μm steps and projecting maximally through the desired tissue unless otherwise noted. To image adult eyes and wings, decapitated heads and dissected wings were imaged with a Canon EOS Rebel camera fitted to a Leica stereo microscope. Individual slices were merged using iSolution-Lite software (IMT-Digital).

Primary antibodies used were: rat α-ELAV (1:50, Developmental Studies Hybridoma Bank [DSHB], 7E8A10); mouse α-Pros (1:100, DSHB, MR1A); rabbit α-GFP (1:2000, Molecular Probes); mouse α-β-galactosidase (1:1000, Promega); guinea pig α-Senseless (1:2000, obtained from H. Bellen, Baylor College of Medicine, Houston, TX, USA); guinea pig α-Salm (1:500, obtained from from Claude Desplan, New York University, New York, NY, USA); mouse α-Cut (1:50, DSHB, 2B10). Secondary antibodies were from Jackson ImmunoResearch: donkey α-rabbit-Cy3 (1:2000), donkey α-rabbit-488 (1:2000), donkey α-rat-Cy3 (1:2000), donkey α-rat-488 (1:2000), donkey α-mouse-Cy3 (1:2000), or donkey α-guinea pig-488 (1:2000). Oregon Green 488 Phalloidin (1,2000, Thermo Fisher Scientific) and DAPI (1,2000, Invitrogen) were used to detect actin and DNA, respectively.

### Quantitative reverse transcription PCR (RT-qPCR)

For each run, 60 pairs of late 3^rd^ instar eye-antennal discs or wing discs were dissected in S2 cell medium, rinsed 1X in PBS, homogenized in 350ul TRIzol (Invitrogen). RNA was extracted from the homogenized sample using Direct-zol RNA Miniprep (Zymo Research) with in-column DNAse treatment. 1 μg RNA was used to carry out reverse transcription using iScript cDNA Synthesis Kit (Bio-Rad Laboratories). qPCR was performed in technical triplicate in 20 μl reactions containing QuantiTect SYBR Green PCR Master Mix (Qiagen), 1 μl cDNA and each primer at 200 nM. Cycling conditions were 95°C for 10 minutes, followed by 40 cycles of denaturation at 95°C for 30 seconds, annealing at 55°C for 30 seconds and extension at 72°C for 30 seconds, and then a final incubation at 95°C for 15 seconds and 60°C for 1 minute. Eukaryotic translation elongation factor 1 alpha 1 (EF1α) was chosen as the reference gene [66], and the average C_T_ was used to analyze the expression levels via the -2^ΔΔCT^ method [67]. The qPCR was performed using iTaq Universal SYBR Green Supermix (Bio-Rad Laboratories) on a 7300 Real-time PCR Machine (Applied Biosystems). Subsequent disassociation analysis was performed with 7300 system software to confirm the sequence specificity of the reaction. Experiments were repeated with independently isolated RNA samples from different disc collections. For statistics, significance was calculated via pair-wise Student T-tests between the mutant indicated versus wildtype. **, p < 0.01; *, p< 0.05.

Primers used:

EF1α-forward 5’GCGTGGGTTTGTGATCAGTTGATCTTCTCCTTGCCCATCC3’, EF1α-reverse 5’GATCTTCTCCTTGCCCATCC3’; *pntP1*-forward 5’CGTGCTGTTGTTGATGCGGT3’, *pntP1*-reverse 5’GACTGGGCTACTTCAATGATAT3’; *pntP2*-forward 5’TCTGTGCAGTTTGTCGGATATT3’, *pntP2*-reverse 5’ACGCGGATCTTTGGTTATGT3’; *pntP3*-forward 5’GCGGATCTTTGGTTATGTTGC3’, *pntP3*-reverse 5’GCAAGCTCAAAGAAGTTCCCAC3’.

### CRISPR/Cas9-mediated generation of *pnt* mutants

To generate *pnt*^*p3*^, a ~2.1 kb fragment including ~1kb upstream and ~1.1kb downstream sequences from the specific start codon was amplified from genomic DNA made from *vas-Cas9* flies, and assembled into the backbone of pHD-Scarless (generated by O'Connor-Giles laboratory, *Drosophila* Genomics Resource Center, 1364; [68]) via Gibson assembly reaction (NEB), and confirmed by sequencing. From the assembled plasmid, a ~1kb fragment (fragment A) containing the downstream sequence from the start codon with stop codons inserted followed by a deletion of 52bp after the start codon, a ~100bp fragment (fragment B) containing the 5’UTR and a ~800bp fragment (fragment C) containing the rest of the upstream sequences were amplified. The transformation marker 3xPax-RFP with PiggyBac (PB) transposase arms (fragment D) and the plasmid backbone (fragment E) were amplified from the pHD-Scarless vector. The five purified fragments were assembled via Gibson assembly reaction. The NGG sequence of the PAM sites in fragment C were mutated via Quikchange Mutagenesis (Stratagene). Guide RNAs were subcloned into the pU6-Bbs1 chiRNA plasmid (Addgene, 45946; [68]). Each template (300 ng/μL) and the two guide RNAs (75 ng/μL), were injected into a GFP/ RFP-negative *vasa-Cas9* strain (a gift from Rick Fehon). G_0_ adults were crossed individually to *w*^*1118*^, and transformants were identified by 3X-Pax-RFP expression in the eyes of the F1 progeny. The eye marker 3XPax-RFP was removed by piggyBac excision by crossing to *Wg*^*Sp*^/ *Cyo*,*Tub>PBac*. RFP-negative progeny were crossed to *TM6B* to establish stocks. The alleles were confirmed by restriction digest and sequencing.

The mutant *pnt*^*p2*^ stain was generated in a similar manner. The cloning scheme of the template was adapted to the positions of the PAM sites on the *pntP2* locus with stop codons inserted at the 202^th^ position of *pntP2*. To generate *pnt*^*p2p3*^, the template and guide RNAs used to generate *pnt*^*p2*^ were injected with *nanos-Cas9* plasmid (a gift from Rick Fehon, [69]) into the *pnt*^*p3*^ strain.

For *pnt*^*p3*^, fragment A-forward 5’ACGATAATACTGGGGCAGGTAAATTTCG3’, reverse 5’CCTGACTATGtaaGGTCGGCAAACTATAAC3’; Fragment B-forward 5’GCCGACCttaCATAGTCAGGCCAATTGAG3’, reverse 5’CTTTCTAGGGTTAAGATCAATTGTACGATCG3’; Fragment C-forward 5’CTAGGGTTAATTGATTGGTGCGGCACAATC3’, reverse 5’GGCCTTTCGCGCGCTGGCTGTTTTATTTG3’; Fragment D-forward 5’CGTACAATTGATCTTAACCCTAGAAAGATAGTCTGCGTAAAATTG3’, reverse 5’CCGCACCAATCAATTAACCCTAGAAAGATAATCATATTGTGACGTACG3’; Fragment E-forward 5’CAGCCAGCGCGCGAAAGGCCCAGTCTTTC3’, reverse 5’ACCTGCCCCAGTATTATCGTTGACATGTATAATTTTGATATCAAAAAC3’; Pam mutation forward 5’CAATCTTGACGCGAAATGTCAGTGA3’, reverse 5’ACATTTCGCGTCAAGATTGTGCCG3’.

For *pnt*^*p2*^, fragment A-forward 5’CATGTCAACGATAATACTGATCAGGCCTTTTGTCTATGC3’, reverse 5’TCTTTCTAGGGTTAAGGCTCAAGAAGAACCGCAAAGTCA3’; fragment B-forward 5’TCTTCTTGAGCCTTAACCCTAGAAAGATAGTCTGC3’, reverse 5’TTAACCCTAGAAAGATAATCATATTGTGACGTACG3’; fragment C-forward 5’TCTTTCTAGGGTTAAttattattaGAGGTGGCTGCTGGCCGGCGAC3’, reverse 5’AGACTGGGCCTTTCGCAAACTAGCCTCGTATCCATAGCT3’; fragment D-forward 5’ATACGAGGCTAGTTTGCGAAAGGCCCAGTCTTTCGA3’, reverse 5’CTGATCAGTATTATCGTTGACATGTATAATTTTGATATCAAAAAC3’.

### Fluorescence In situ hybridization (FISH)

DNA oligos were designed through Stellaris® RNA FISH (https://www.biosearchtech.com/stellaris-designer), with 34 DNA probes targeting *pntP1* and 36 targeting *pntP2*. Given the short specific exon of *pntP3*, we could design at most 23 specific probes; using that set, we were not able to detect *pntP3* transcript in the discs. DNA oligos were ordered from Integrated DNA Technologies with an amine modification at the 5’ end. 13.5 μL of 100 μM DNA was mixed with 1.5 μL of 1 M NaHCO_3_ (pH 8.6) and 25 μg of NHS ester-ATT0 633 fluorophore (Lumiprobe) dissolved in 0.5 μL DMSO. The mixture was incubated overnight at 37°C. Conjugated DNA oligo was precipitated by adding 1.67 μL of 3 M NaOAc and 50 μL of 100% ethanol overnight at −20°C. The precipitated DNA oligo was pelleted by centrifugation for 30 min at 21000 g and re-suspended in 40 μL water. The DNA solution was passed through a Microspin G-25 column (GE Healthcare) to remove any residual free dye and salt. The overall fluorophore labeling efficiency was ~50%.

White prepupal eye discs were dissected in cold S2 cell medium, fixed for 15 min in 1% PFA, incubated in methanol at room temperature for 30 min, washed 1X in wash buffer (4X SSC, 0.1% Tween-20). DNA probes were diluted in hybridization buffer (10% dextran sulfate, 4X SSC, .01% (wt/vol) salmon sperm ssDNA, 1% vanadyl ribonucleoside, 0.2mg/mL BSA, 0.1% Tween-20) at 1:20 ratio, preheated at 62°C for 10 min. Batches of three eye discs were incubated with 100 μL diluted probes at 62°C for 1 hr, washed 1X in wash buffer at 62°C for 5 min, incubated in PTW (PBS, 0.1% Tween-20) with DAPI (1,2000, Invitrogen) at room temperature for 10 min. Discs were mounted in 15ul Vectashield (Vectorlabs) and imaged with a Zeiss LSM 880 confocal microscope, using 0.8 μm steps and projecting maximally through the tissue. In all pair-wise comparison of wild type vs. *pnt* mutant, discs from the two strains were dissected, processed and imaged in parallel.

For quantification of signal intensity, individual fluorescent spots were identified from maximum projection images taken using the 633 emission channel, with the same thresholding applied across images from the same batch. The resulting punta were analyzed with Fiji ImageJ. For each spot, mean grey value, the x-axis of the centroid and area size in pixel unit were extracted. The relative number of transcripts in each spot was represented by the cumulative grey value, which was calculated by area size multiplied by mean grey value. Outliers (top 5% of the transcript numbers of each image) were excluded. X-axis values for each image was calibrated based on distance from the MF. Total transcripts per pixel on the x-axis was calculated as the sum of grey values of spots grouped into one-pixel window on the x-axis. The average transcript per spot on the x-axis plotted in S6 Fig was the sum of grey values of spots divided by the count of spots in each one-pixel window.

## Supporting information

S1 Fig Genomic organization and conservation of *pnt*. **(A)** A screen shot of the *pnt* locus from Flybase G-Browse, oriented 5’ to 3’ from right to left, summarizing the annotation of the B, C, D and E isoforms. **(B, C)** PntP2 (B) and PntP3 (C) are conserved from *D*. *melanogaster* (*) to *D*. *virilis* (**). Alignments show the unique N-terminal sequences (within the red rectangle) of PntP2 and PntP3. Conserved sequence begins immediately N-terminal to the PLTP (blue line)/SAM region (unboxed). The PntP2 N-terminus is 62% identical and 70.7% similar between *D*. *melanogaster* and *D*. *virilis*, while the PntP3 N-termini are 57% identical and 63% similar.
(PDF)

S2 Fig Overexpressed PntP3 disrupts eye development more strongly than overexpressed PntP2. All images show maximum projections of optical confocal sections of representative 3^rd^ instar eye imaginal discs, oriented anterior left. Scale bars: 10 μm. **(A-C)** Overexpression of *UAS*-*pntP2* and *UAS*-*pntP3*, driven by *lz-Gal4*. Elav (red) marks all photoreceptors and Pros (green) marks R7 photoreceptors and cone cells. (A’-C’) Overexpression of *pntP3* induced more ectopic Pros-positive cells than overexpression of *pntP2*. (A”-C”) Magnified views of boxed regions in A’-C’ with further zoom in to a single ommatidium (red box, red arrow). The wild type pattern of four Pros positive cone cells is labeled in 2A”. **(D-I)** Overexpression of *UAS-pntP2* and *UAS-pntP3*, driven by *ro-Gal4*. Elav (red) marks the photoreceptors and DAPI (blue) marks all nuclei. (D-F) Overexpression of *pntP3* induced more ectopic Elav expression than overexpression of *pntP2*. (D’-F’) Magnified views of boxed regions in D-F. (G-I) Staining with anti-Flag to detect the epitope tag shows comparable levels and nuclear localization of PntP2 and PntP3.
(PDF)

S3 Fig PntP3 is expressed in R2-R5 photoreceptors and cone cells. **(A)** Higher magnification and single optical slice showed the overlapping expression patterns of *pnt*^*1277*^ (β-gal, red) and GFP-PntP3 (GFP, green) in cone cells (as indicated by the yellow arrows). Scale bar: 10 μm. **(B)** Moving averages of GFP-PntP3 levels highlight the peaks of expression at the MF (orange arrowhead) region in the first five photoreceptors recruited to each ommatidium, R8, R2, R5, R3 and R4 (pink, dark blue, and orange lines). Expression in photoreceptors specified after the SMW, R1, R6, and R7, was not above baseline (light blue and purple lines. A slow increase in PntP3 levels was measured in the cone cells (green line). Data plotted are from two discs from independent experiments and show the results from scoring 404 ommatidia with 404 R8 cells. Single cell measurements were made and plotted as described in [70].
(PDF)

S4 Fig Cone cell loss in *pnt*^*p2*^ mutants. **(A-B)** Representative third instar eye discs oriented anterior left, stained with anti-Cut to mark the cone cells. Homozygous *pnt*^*p3*^ mutants appear fully wild type (A) while only a few scattered Cut-positive cells remain in *pnt*^*p2*^ mutants (B). Scale bar: 10 μm.
(PDF)

S5 Fig *pntP1* transcription did not change in *pnt*^*p2*^ and *pnt*^*p3*^ mutant wing imaginal discs. **(A)** RT-qPCR comparison of *pntP1* transcript levels in wild type versus *pnt*^*p2*^ (blue bars) and *pnt*^*p3*^ (orange bars) null mutant 3^rd^ instar wing imaginal discs. No significant change was detected. Error bars represent standard deviations of three independent experiments. (B) Quantification of *pntP1* FISH in the wildtype disc of Fig 4B from maximum projections. *pntP2* levels begin to rise to peak at the MF (yellow arrow), quickly decrease between MF and SMW (red arrow) and slowly decrease to a steady state posterior to the SMW. Each dot plots the product of the fluorescent intensity and the size of an individual *pntP1* FISH focus, representing the relative amount of *pntP1* transcript (y-axis on the left) The line connects the moving average of the sum of all foci within one-pixel windows along the x-axis (y-axis on the right).
(PDF)

S6 Fig *pntP2* transcriptional autoregulation. **(A)** Single optical slice from a 3^rd^ instar eye disc shows individual *pntP2* FISH foci (white dots) in each cell. DAPI (magenta) marks the nuclei. **(B-C)** Additional quantitative analysis of pntP2 FISH in wild type (green dots) versus *pnt*^*p2*^ mutants (red triangles) using maximal projections of the same set of eye imaginal discs used for the analysis in Fig 5E and 5F. **(B)** Each dot/triangle plots the average *pntP2* transcript per cell (quantified as the product of focus intensity and area) for each pixel window along the x-axis. Error bars depict standard deviation. Consistent with the data and analysis in Fig 5C–5F, the average *pntP2* transcript level per cell is higher in the peak region and lower in the posterior in *pnt*^*p2*^ mutant discs than in corresponding regions in wild type discs. (**C**) Quantification of the number of *pntP2* FISH foci counted. The dots/triangles represent the total number of foci counted for each pixel window along the x-axis. In *pnt*^*p2*^ mutant discs, more foci were counted in cells leading up to the peak region and fewer were counted in the posterior (blue arrow and to the right). Together with S6B Fig, this analysis suggests that the increase in *pntP2* transcript in *pnt*^*p2*^ mutant is a compound effect of increased transcription in cells normally transcribing *pntP2* at a detectable level, and increased transcription in cells that normally transcribe *pntP2* below a detectable level. Conversely, decreased transcription in cells in the posterior results in both lower average transcript per cell and fewer cells with detectable levels.
(PDF)

S7 Fig Combining genetic with temperature stress reveals contributions of *pntP2* and *pntP3* to developmental robustness. **(A-B)** Single optical slice of phalloidin staining of representative adult eyes of indicated genotype. The animals had been subjected to temperature stress during larval development. *egfr/rl* (A), *egfr/rl; pnt*^*p3*^/*pnt*^*p2*^ (B). Red arrow points to an ommatidium lacking the central R7 rhabdomere and yellow star points to an ommatidium that also lost outer R1-R6 rhabdomeres. Scale bars: 5 μm. **(C)** Quantification of photoreceptor loss in phalloidin-stained adult eyes expressed as the frequency of ommatidia with all seven rhabdomeres (R1-R7), losing R7 only, losing one of the outer R1-R6, or losing both R7 and more than one outer rhabdomeres. n represents the number of ommatidia scored for each genotype. In the *egfr/rl* controls, more than 99% ommatidia had all R1-R7 rhabdomeres. Heterozygosity for either *pnt*^*p3*^ or *pnt*^*p2*^ or homozygous loss of *pnt*^*p3*^ were essentially indistinguishable from control. In contrast, in *egfr/rl; pnt*^*p3*^/*pnt*^*p2*^ retinas, only 88% of ommatidia had the full complement of R1-R7 rhabdomeres. Of the 12% that lost photoreceptors, 7% lost only R7, 2% lost only one outer, 1% lost both R7 and one outer and 1% lost more than one outer. Significance was calculated via two tailed Student T-tests between each mutant and the *egfr/rl* control. Only *egfr/rl; pnt*^*p3*^/*pnt*^*p2*^ showed significant photoreceptor loss, ****, p < 0.0001.
(PDF)

S8 Fig Reducing the dose of *pnt*^*p3*^ and *pnt*^*p2*^ enhances *Sev-Yan*^*act*^ induced photoreceptor loss. **(A-B)** Phalloidin staining of representative adult eyes of indicated genotype showing that homozygous *pnt*^*p3*^ (A) or heterozygous *pnt*^*p2p3*^ (B) enhances the *Sev-Yan*^*act*^ induced photoreceptor loss. Scale bar: 5 μm.
(PDF)

S1 Table Source Data. (XLSX)
