# Supplementary material for: A context-dependent bifurcation in the Pointed transcriptional effector network contributes specificity and robustness to retinal cell fate acquisition
Source: PLoS Genet. 2020 Nov 30;16(11):e1009216. doi: 10.1371/journal.pgen.1009216 (PMC7728396; doi:10.1371/journal.pgen.1009216)
Supplement: S1 Fig — (A) A screen shot of the pnt locus from Flybase G-Browse, oriented 5’ to 3’ from right to left, summarizing the annotation of the B, C, D and E isoforms. (B, C) PntP2 (B) and PntP3 (C) are conserved from D. melanogaster (*) to D. virilis (**). Alignments show the unique N-terminal sequences (within the red rectangle) of PntP2 and PntP3. Conserved sequence begins immediately N-terminal to the PLTP (blue line)/SAM region (unboxed). The PntP2 N-terminus is 62% identical and 70.7% similar between D. melanogaster and D. virilis, while the PntP3 N-termini are 57% identical and 63% similar. (PDF) [file pgen.1009216.s001.pdf]

A

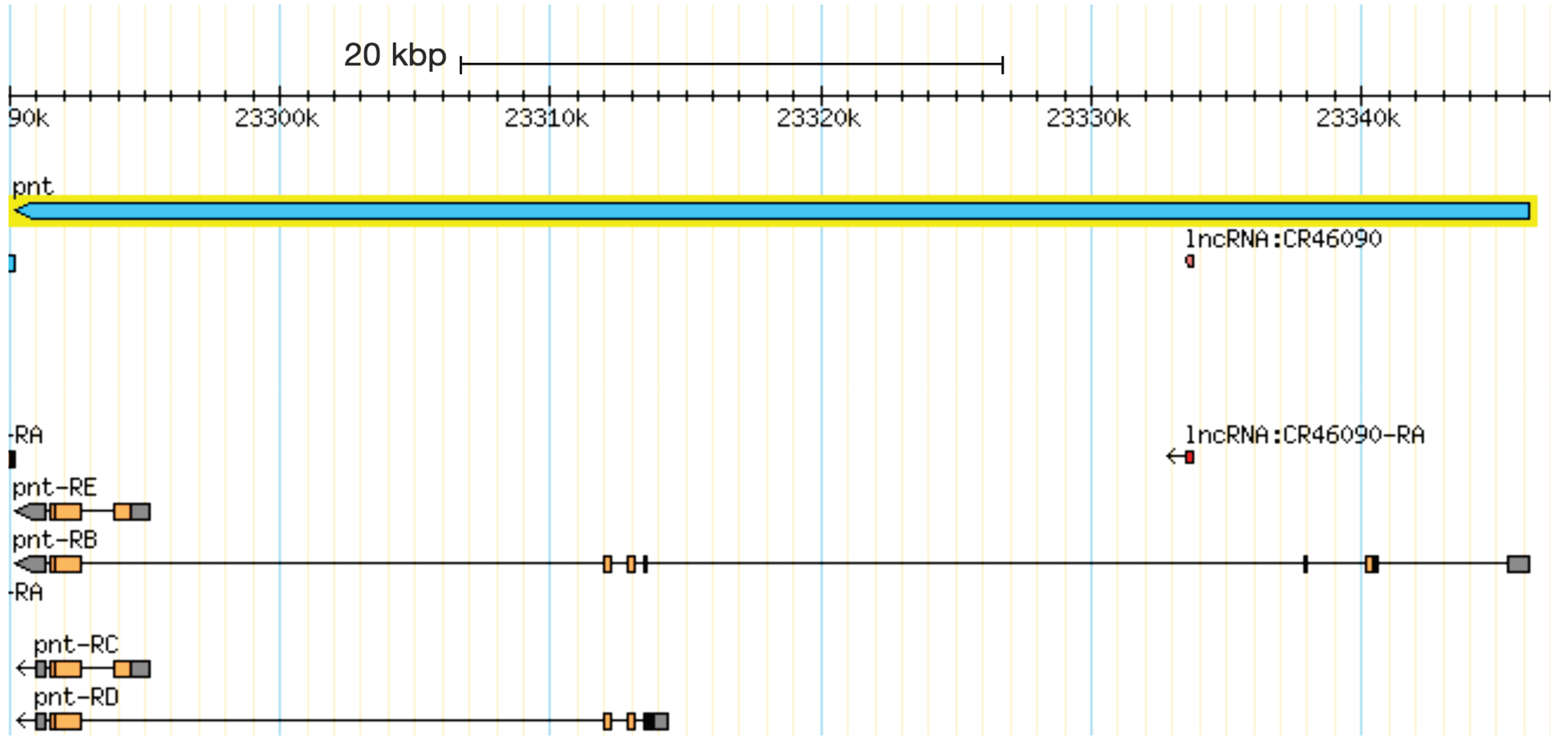

B

```
  P2_Dananassae       MELAICKTDLSATKFMLPPALPASAAIGTTSAAAATVSSHSHSHSTAAAAQLAFLDKAAH   60
* P2_Dmel             MELAICKTDLSATKFMLPPALPSSAAIGSSSAVAST--------------ASHFLDKAAH   46
  P2_Dyakuba          MELAICKTDLSATKFMLPPALPTSATIGSTSAVAST--------------ASHFLGKATH   46
  P2_Dpseudoobscura   MELAICKTDLSATKFMLPPPALPPQATAIATGGPDHSASSVA--------QFSF-HHNLN   51
**P2_Dvirilis         MEVAICKTDLSATKFMLPPALPAAAAIATTTATA-TTH-SF----------LDR-AVHFN   47
  P2_Dmojavensis      MELAICKTDLSATKFMLPPALPAAAAIATTTATATATH-SF----------LDR-AAQIN   48

  P2_Dananassae       ELFNLNAINGHLFKPPPSHHTNSSNSA-------------ANSSSQASTMRLKKNRKVTF   107
* P2_Dmel             ELFQLNAINGHLFKSPASSHLNSVGSP-SILSQLNGIGNSGNHSGQVSTMRLKKNRKVTF   105
  P2_Dyakuba          EFIHLNAINGHLFKSPATNHHNSLGSPQSILSQLNGIGNSSSHSGQVSTMRLKKNRKVTF   106
  P2_Dpseudoobscura   ELFNGFS-H----NTLCH--------NSTFSIHPSVVPSILNSNNTTSNMRLKKNRKVTF   98
**P2_Dvirilis         ELLNFNA-GQHLFKTSCN--------PSSFLNNS--SSSSNCSSSSSSNMRLKKNRKVTF   96
  P2_Dmojavensis      ELFNFNP-GQHLFKTNN-----------SFLNNN--NSSS---NSSSSNMRLKKNRKVTF   91

  P2_Dananassae       LSSIVESKTIFIKEEPIHGCKDLPPPICSLSDISDHEASLDVPTALPPLTPGTNRKVNEV   167
* P2_Dmel             LSSLVESKNIFIKEEPIHGCKD----LCSLSDISDHEASLEVPTALPPLTPGTNRKVNEV   161
  P2_Dyakuba          LSSLVESKNIFIKEEPIHGCKD----LCSLSDISDQEASLEVPTTLPPLTPGTNRKVNEV   162
  P2_Dpseudoobscura   LSSLVESKNIFIKEEPINGCKDL-------SDISDHEASLDVPTALPPLTPGTNRKVNEV   151
**P2_Dvirilis         LSSLVESTTKYIKEEPVNGCKDLT--VCSLSDISDHEASLDVPTTLPPLTPGTNRKVNEV   154
  P2_Dmojavensis      LSSIVESTTKYIKEEPVNGCKDLP--VCSLSDISDHEASLDVPTALPPLTPGTNRKVNEV   149
```

C

```
  P3_Dananassae       MTNEWIDWNDSRMLPPLRSANYNHHP-------------------STFLNNNYHSFSSKF   41
* P3_Dmel             MTNEWIDWNDSRMLPPLRSANYNYHP-------------------QTFLPNNYQCFTGKF   41
  P3_Dyakuba          MTNEWIDWNDSRMLPPLRSANYNHHH-------------------QTFLPNNYQCFTGKF   41
  P3_Dpseudoobscura   MTNEWIDWSDSRMLPPLRSAHYNHHHHHHQTLLQNCNNNSSSNNNNNNNNNSYHSFSSKF   60
**P3_Dvirilis         MTNEWIDWNDCRMLPPLRSANYNTNNNNNSNSNINSNQSNYNNNHQTFLSNNYQCLTSKF   60
  P3_Dmojavensis      MTNEWIDWNDCRMLPPLRSANYNSNSNN--------NNNNYNNNHQTILSNSYQSLTSKF   52

  P3_Dananassae       HLKAQKLQQLSTNHPHKLKDVPTALPPLTPGTNRKVNEVLKASFASWEKEVQNCNITKDP   101
* P3_Dmel             HLKGQKLQQLTTNHS-KLKEVPTALPPLTPGTNRKVNEVLKASFASWEKEVQKCNITKDP   100
  P3_Dyakuba          HLKGQKLQQLTTNHS-KLKEVPTTLPPLTPGTNRKVNEVLKASFASWEKEVQKCNITKDP   100
  P3_Dpseudoobscura   HLKAHKLQQLSTNHS-RLKDVPTALPPLTPGTNRKVNEVLKASFASWEKEVQKYNITKDP   119
**P3_Dvirilis         HLKAHKLQQLHTSNG-RLRDVPTTLPPLTPGTNRKVNEVLKASFASWEKEVQNCNITKDP   119
  P3_Dmojavensis      HLKAHKLQQLHTSNG-KLRDVPTALPPLTPGTNRKVNEVLKASFASWEKEVQNCNITKDP   111
```
