# Supplementary figures and images for: A context-dependent bifurcation in the Pointed transcriptional effector network contributes specificity and robustness to retinal cell fate acquisition
Source: PLoS Genet. 2020 Nov 30;16(11):e1009216. doi: 10.1371/journal.pgen.1009216 (PMC7728396; doi:10.1371/journal.pgen.1009216)

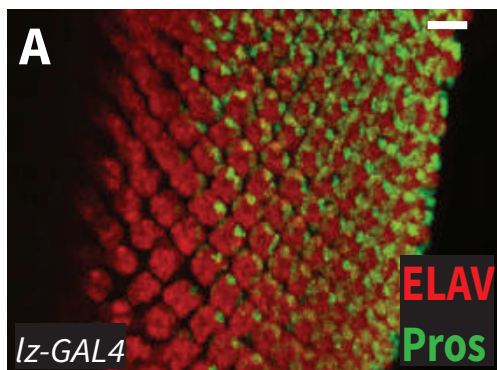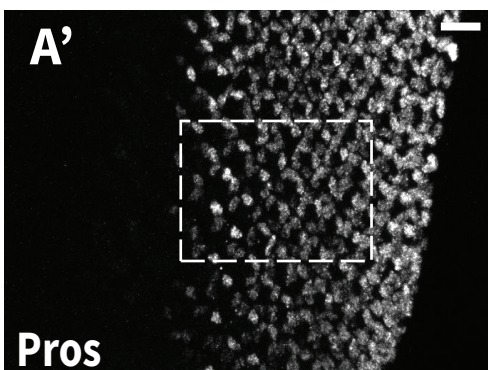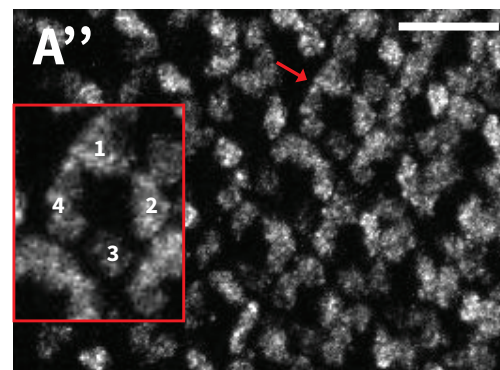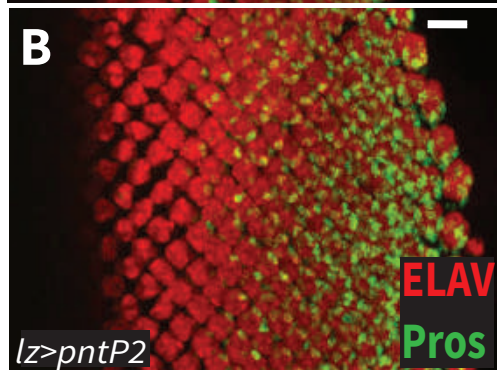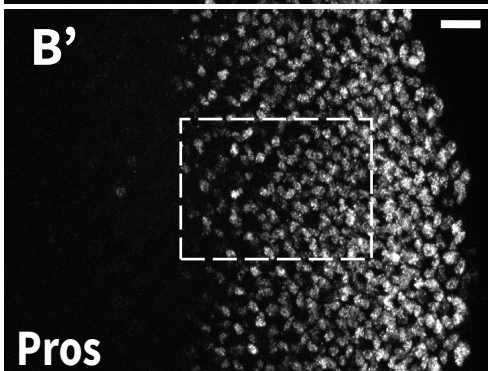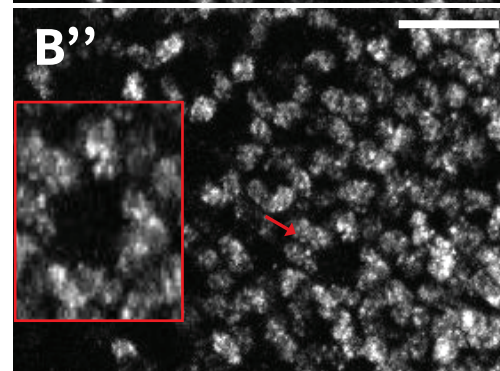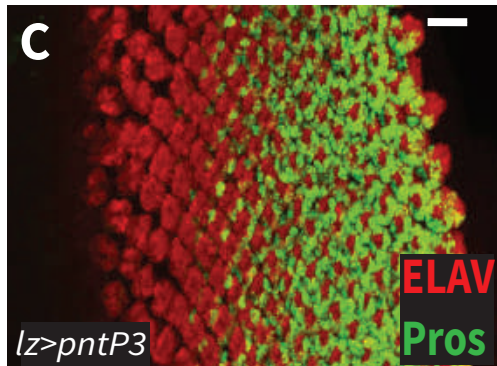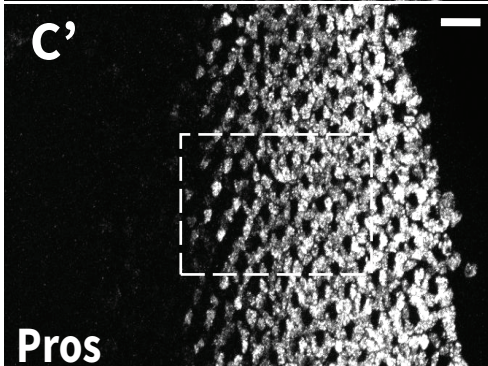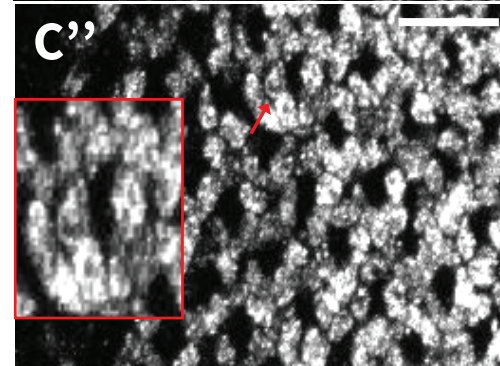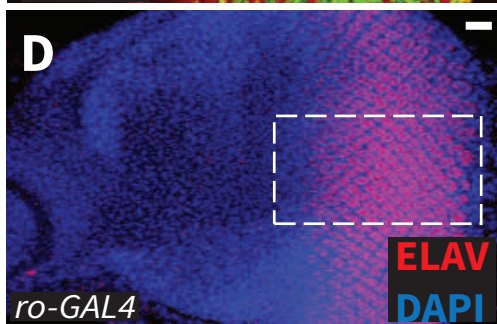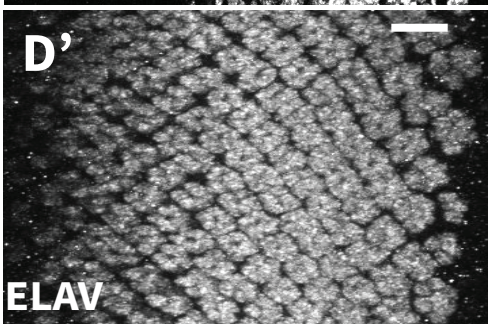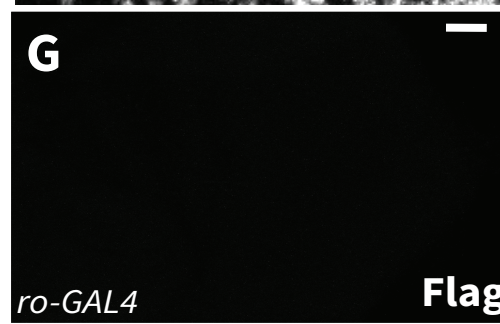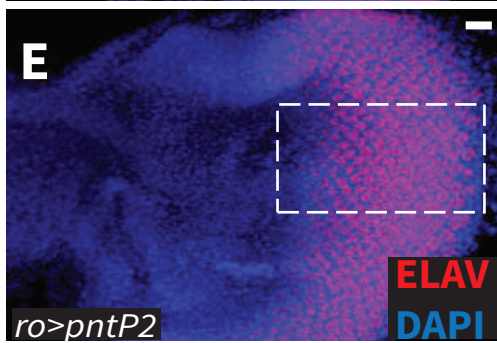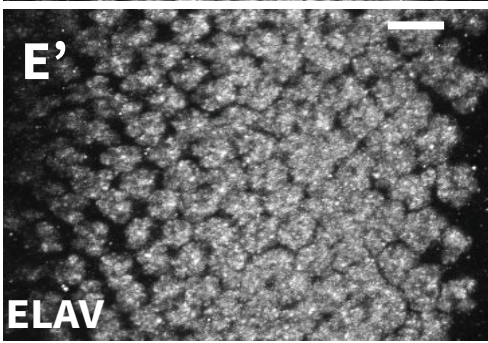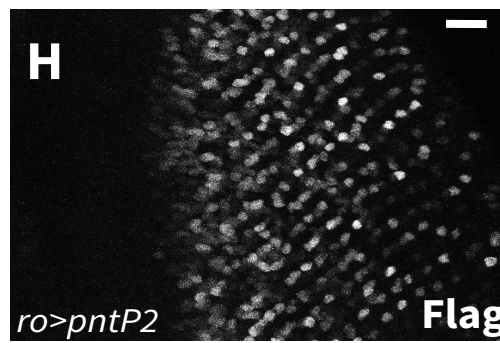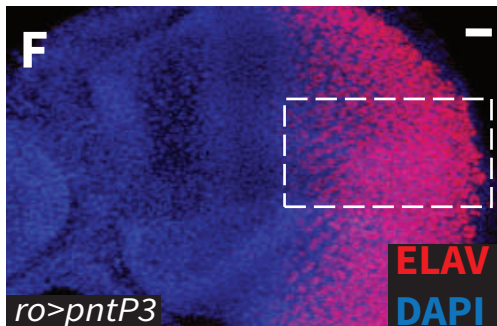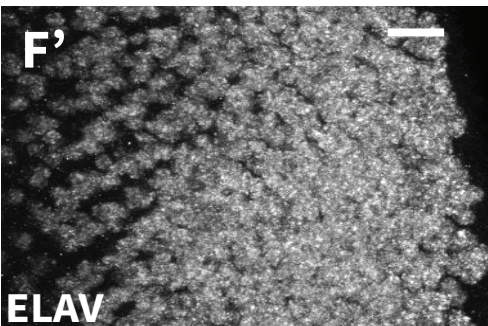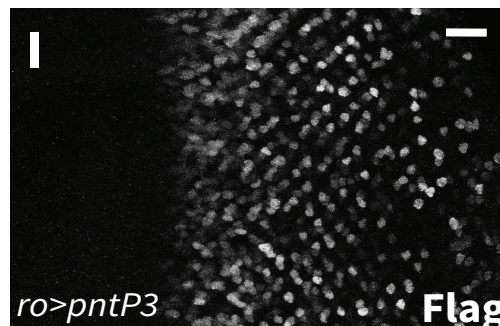

Supplement: S2 Fig — All images show maximum projections of optical confocal sections of representative 3rd instar eye imaginal discs, oriented anterior left. Scale bars: 10 μm. (A-C) Overexpression of UAS-pntP2 and UAS-pntP3, driven by lz-Gal4. Elav (red) marks all photoreceptors and Pros (green) marks R7 photoreceptors and cone cells. (A’-C’) Overexpression of pntP3 induced more ectopic Pros-positive cells than overexpression of pntP2. (A”-C”) Magnified views of boxed regions in A’-C’ with further zoom in to a single ommatidium (red box, red arrow). The wild type pattern of four Pros positive cone cells is labeled in 2A”. (D-I) Overexpression of UAS-pntP2 and UAS-pntP3, driven by ro-Gal4. Elav (red) marks the photoreceptors and DAPI (blue) marks all nuclei. (D-F) Overexpression of pntP3 induced more ectopic Elav expression than overexpression of pntP2. (D’-F’) Magnified views of boxed regions in D-F. (G-I) Staining with anti-Flag to detect the epitope tag shows comparable levels and nuclear localization of PntP2 and PntP3. (PDF) [file pgen.1009216.s002.pdf]

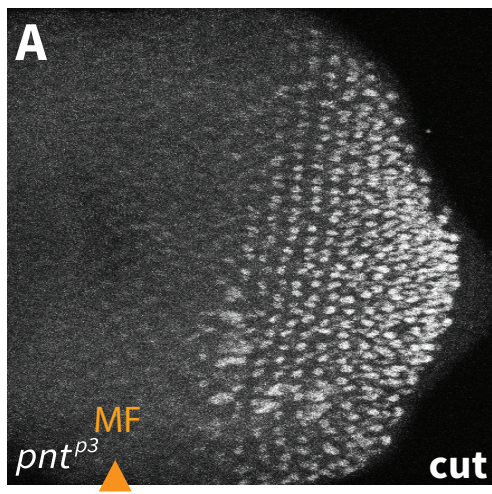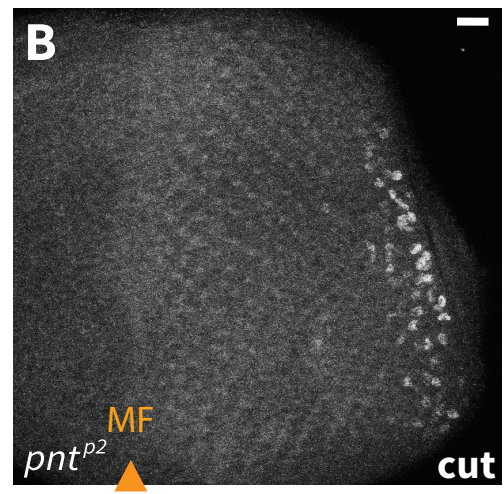

Supplement: S4 Fig — (A-B) Representative third instar eye discs oriented anterior left, stained with anti-Cut to mark the cone cells. Homozygous pntp3 mutants appear fully wild type (A) while only a few scattered Cut-positive cells remain in pntp2 mutants (B). Scale bar: 10 μm. (PDF) [file pgen.1009216.s004.pdf]

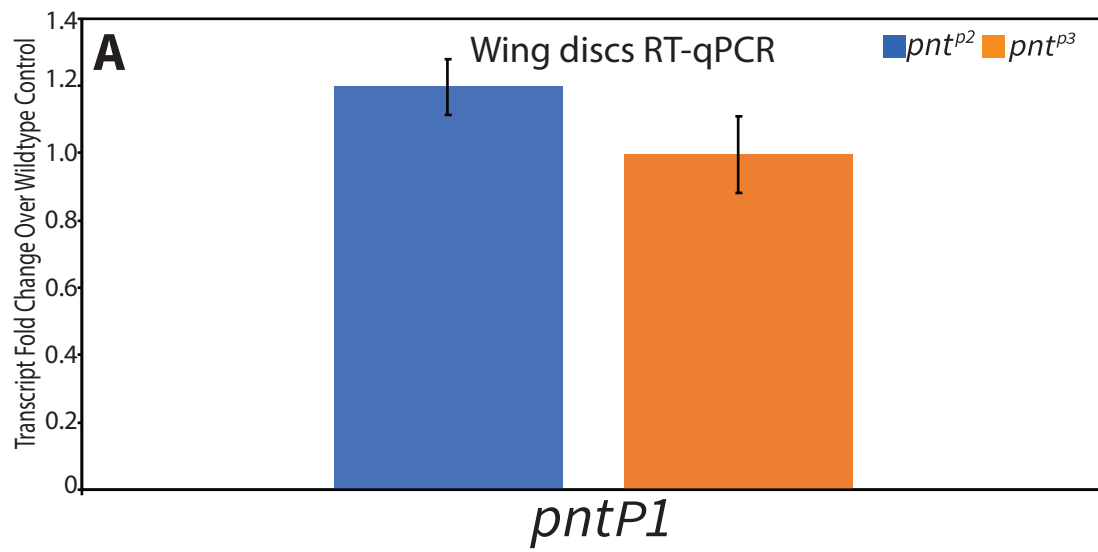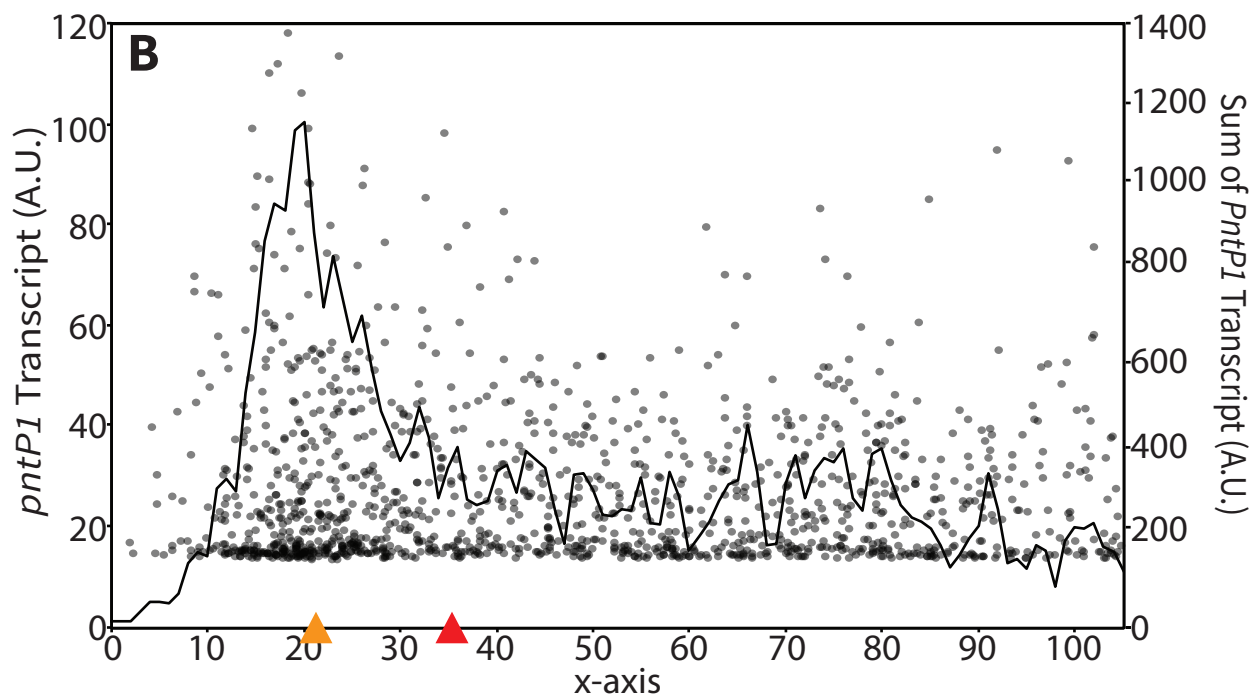

Supplement: S5 Fig — (A) RT-qPCR comparison of pntP1 transcript levels in wild type versus pntp2 (blue bars) and pntp3 (orange bars) null mutant 3rd instar wing imaginal discs. No significant change was detected. Error bars represent standard deviations of three independent experiments. (B) Quantification of pntP1 FISH in the wildtype disc of Fig 4B from maximum projections. pntP2 levels begin to rise to peak at the MF (yellow arrow), quickly decrease between MF and SMW (red arrow) and slowly decrease to a steady state posterior to the SMW. Each dot plots the product of the fluorescent intensity and the size of an individual pntP1 FISH focus, representing the relative amount of pntP1 transcript (y-axis on the left) The line connects the moving average of the sum of all foci within one-pixel windows along the x-axis (y-axis on the right). (PDF) [file pgen.1009216.s005.pdf]

**A**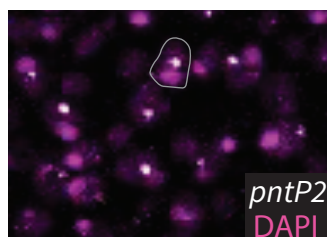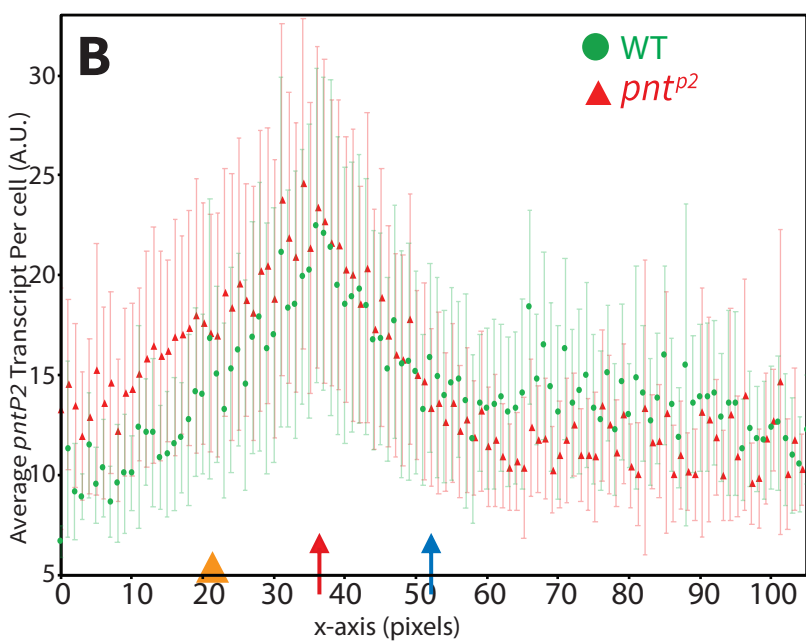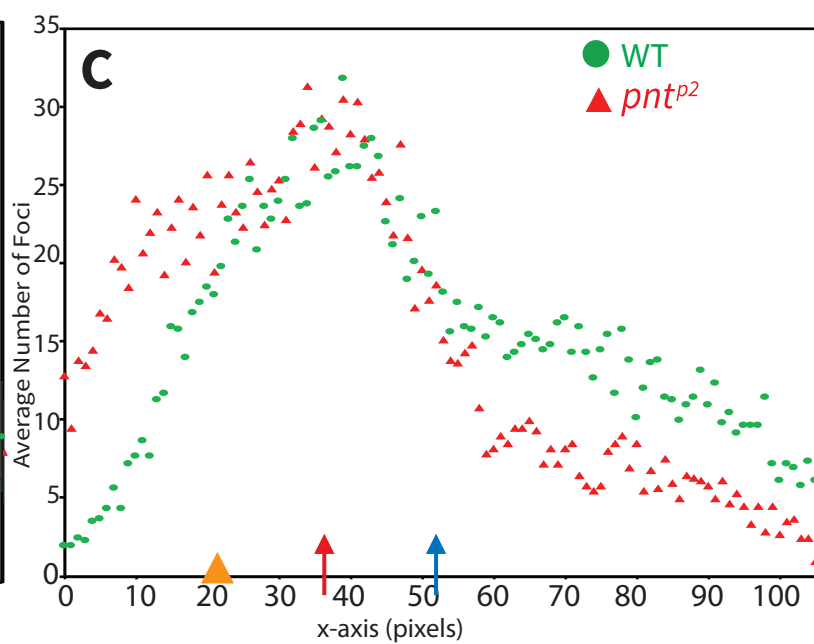

Supplement: S6 Fig — (A) Single optical slice from a 3rd instar eye disc shows individual pntP2 FISH foci (white dots) in each cell. DAPI (magenta) marks the nuclei. (B-C) Additional quantitative analysis of pntP2 FISH in wild type (green dots) versus pntp2 mutants (red triangles) using maximal projections of the same set of eye imaginal discs used for the analysis in Fig 5E and 5F. (B) Each dot/triangle plots the average pntP2 transcript per cell (quantified as the product of focus intensity and area) for each pixel window along the x-axis. Error bars depict standard deviation. Consistent with the data and analysis in Fig 5C–5F, the average pntP2 transcript level per cell is higher in the peak region and lower in the posterior in pntp2 mutant discs than in corresponding regions in wild type discs. (C) Quantification of the number of pntP2 FISH foci counted. The dots/triangles represent the total number of foci counted for each pixel window along the x-axis. In pntp2 mutant discs, more foci were counted in cells leading up to the peak region and fewer were counted in the posterior (blue arrow and to the right). Together with S6B Fig, this analysis suggests that the increase in pntP2 transcript in pntp2 mutant is a compound effect of increased transcription in cells normally transcribing pntP2 at a detectable level, and increased transcription in cells that normally transcribe pntP2 below a detectable level. Conversely, decreased transcription in cells in the posterior results in both lower average transcript per cell and fewer cells with detectable levels. (PDF) [file pgen.1009216.s006.pdf]

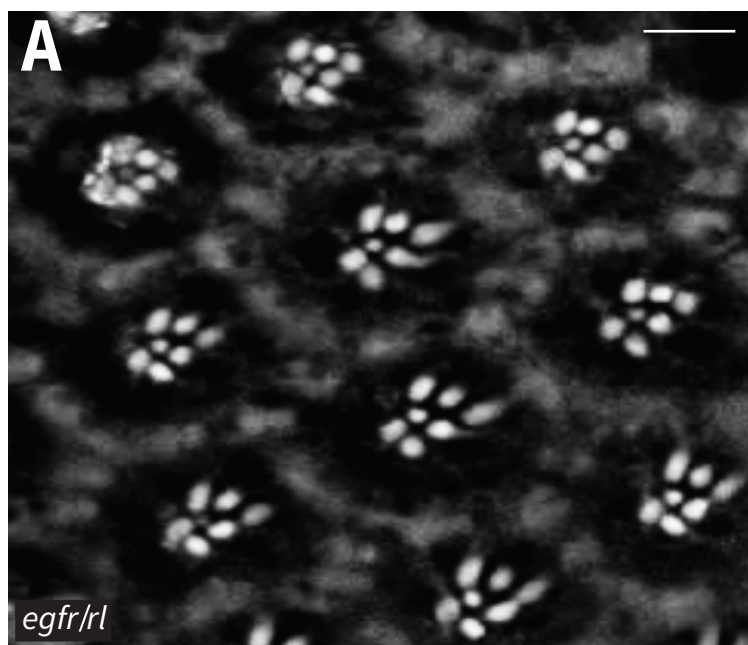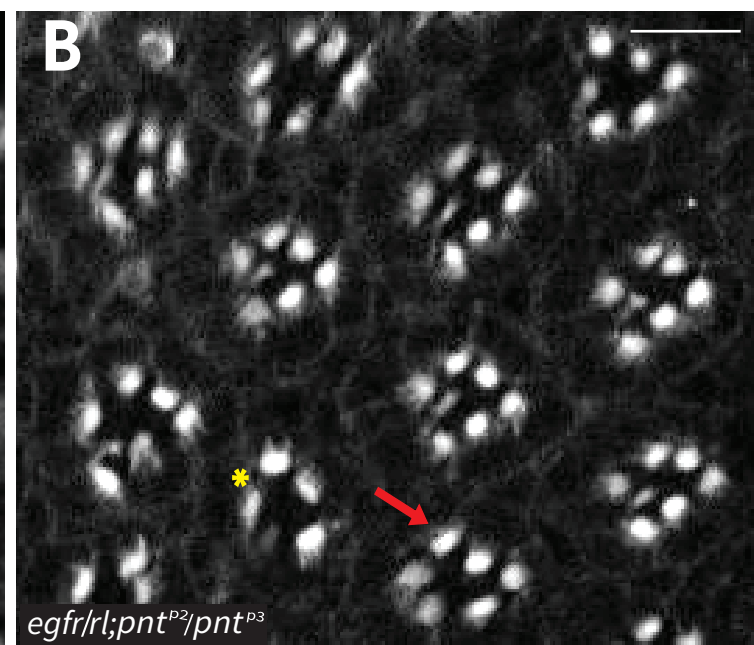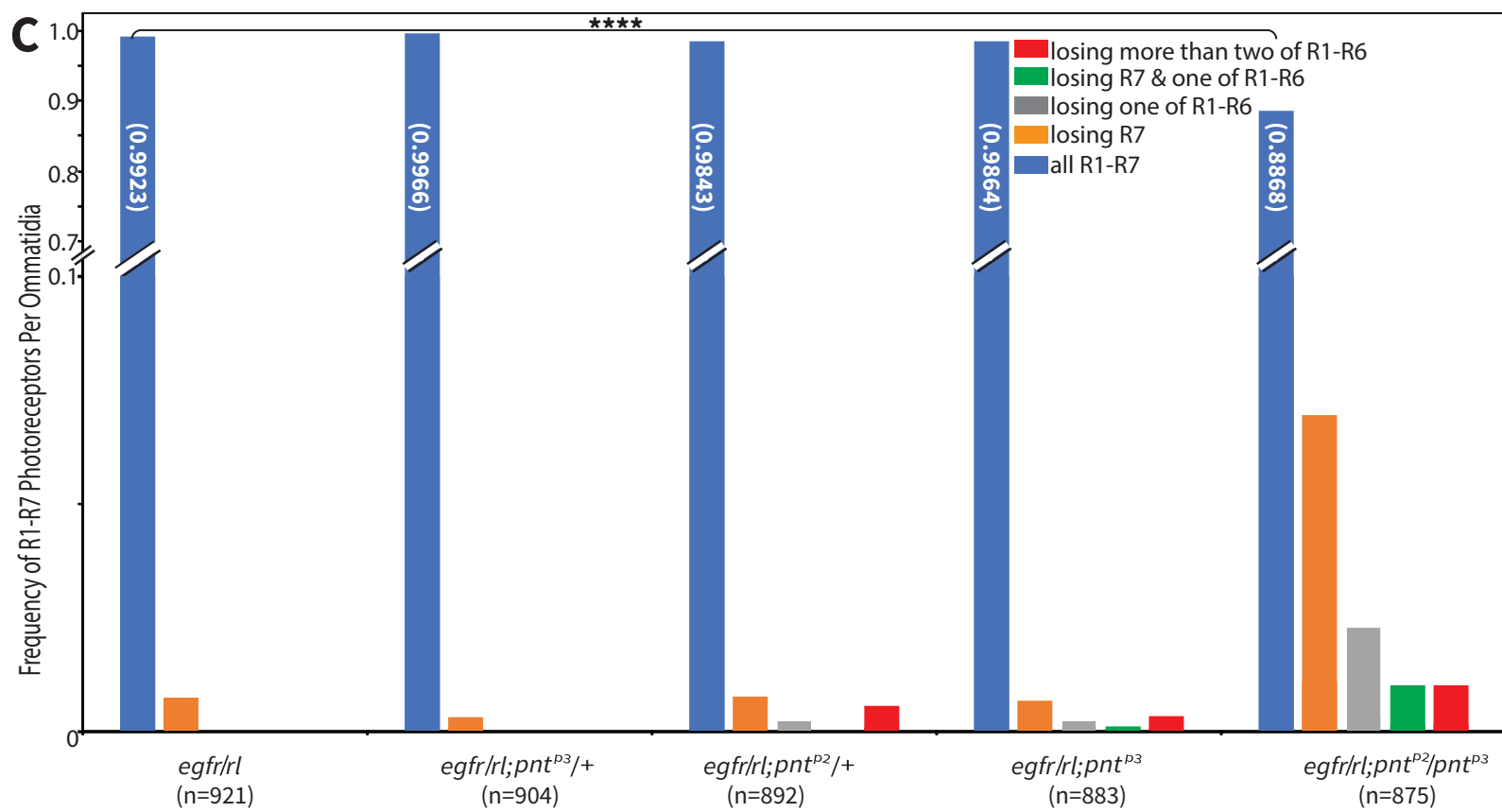

Supplement: S7 Fig — (A-B) Single optical slice of phalloidin staining of representative adult eyes of indicated genotype. The animals had been subjected to temperature stress during larval development. egfr/rl (A), egfr/rl; pntp3/pntp2 (B). Red arrow points to an ommatidium lacking the central R7 rhabdomere and yellow star points to an ommatidium that also lost outer R1-R6 rhabdomeres. Scale bars: 5 μm. (C) Quantification of photoreceptor loss in phalloidin-stained adult eyes expressed as the frequency of ommatidia with all seven rhabdomeres (R1-R7), losing R7 only, losing one of the outer R1-R6, or losing both R7 and more than one outer rhabdomeres. n represents the number of ommatidia scored for each genotype. In the egfr/rl controls, more than 99% ommatidia had all R1-R7 rhabdomeres. Heterozygosity for either pntp3 or pntp2 or homozygous loss of pntp3 were essentially indistinguishable from control. In contrast, in egfr/rl; pntp3/pntp2 retinas, only 88% of ommatidia had the full complement of R1-R7 rhabdomeres. Of the 12% that lost photoreceptors, 7% lost only R7, 2% lost only one outer, 1% lost both R7 and one outer and 1% lost more than one outer. Significance was calculated via two tailed Student T-tests between each mutant and the egfr/rl control. Only egfr/rl; pntp3/pntp2 showed significant photoreceptor loss, ****, p < 0.0001. (PDF) [file pgen.1009216.s007.pdf]

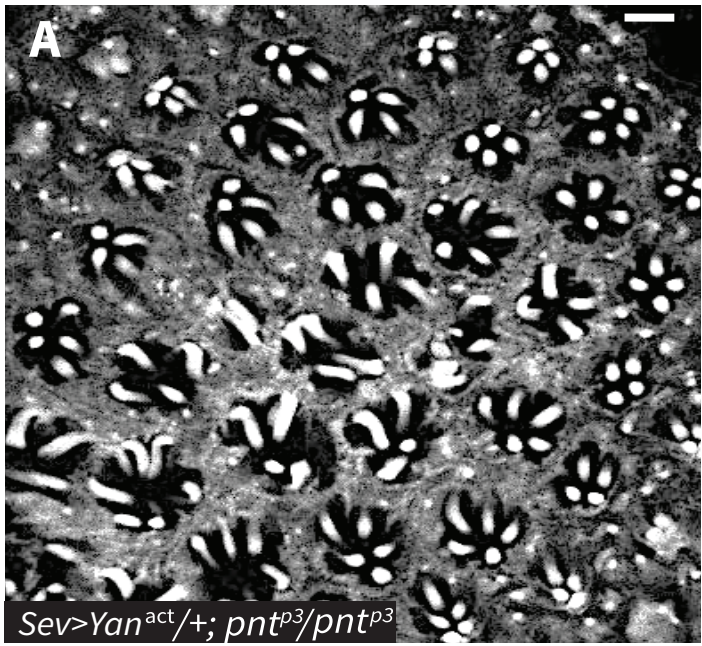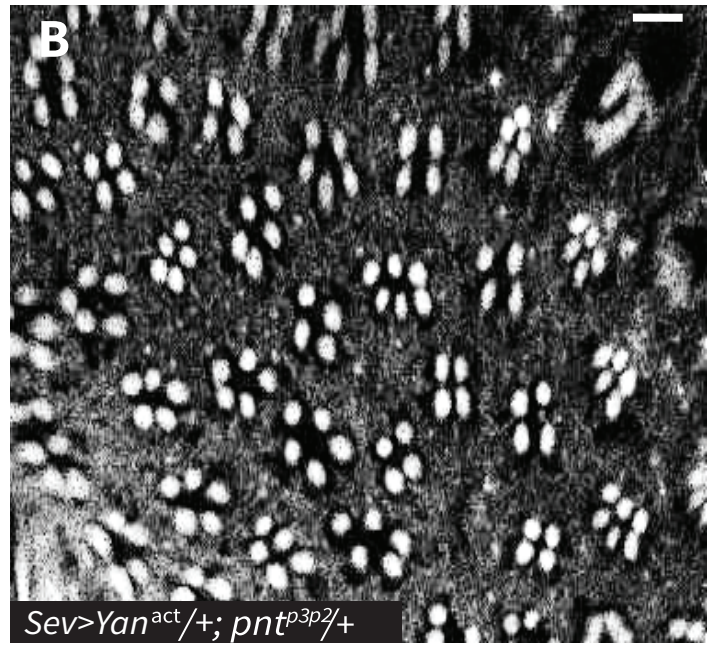

Supplement: S8 Fig — (A-B) Phalloidin staining of representative adult eyes of indicated genotype showing that homozygous pntp3 (A) or heterozygous pntp2p3 (B) enhances the Sev-Yanact induced photoreceptor loss. Scale bar: 5 μm. (PDF) [file pgen.1009216.s008.pdf]
