# Supplementary material for: A context-dependent bifurcation in the Pointed transcriptional effector network contributes specificity and robustness to retinal cell fate acquisition
Source: PLoS Genet. 2020 Nov 30;16(11):e1009216. doi: 10.1371/journal.pgen.1009216 (PMC7728396; doi:10.1371/journal.pgen.1009216)
Supplement: S3 Fig — (A) Higher magnification and single optical slice showed the overlapping expression patterns of pnt1277 (β-gal, red) and GFP-PntP3 (GFP, green) in cone cells (as indicated by the yellow arrows). Scale bar: 10 μm. (B) Moving averages of GFP-PntP3 levels highlight the peaks of expression at the MF (orange arrowhead) region in the first five photoreceptors recruited to each ommatidium, R8, R2, R5, R3 and R4 (pink, dark blue, and orange lines). Expression in photoreceptors specified after the SMW, R1, R6, and R7, was not above baseline (light blue and purple lines. A slow increase in PntP3 levels was measured in the cone cells (green line). Data plotted are from two discs from independent experiments and show the results from scoring 404 ommatidia with 404 R8 cells. Single cell measurements were made and plotted as described in [70]. (PDF) [file pgen.1009216.s003.pdf]

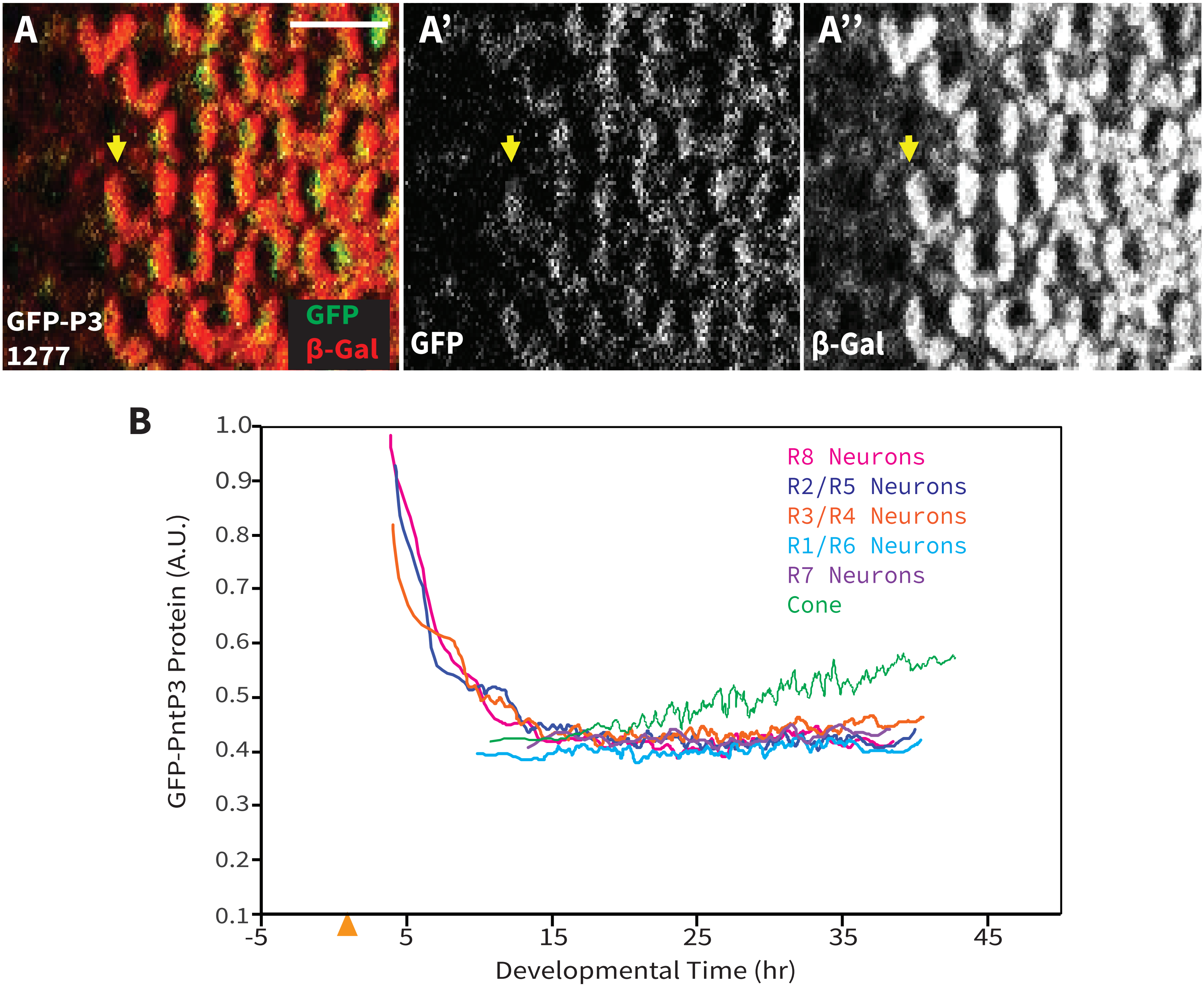

References

1. Nicola´s Pela´ez, Arnau Gavalda-Miralles, Bao Wang, Heliodoro Tejedor Navarro, Herman Gudjonson, Ilaria Rebay, Aaron R Dinner, Aggelos K Katsaggelos, Lui´s AN Amaral RWC. Dynamics and heterogeneity of a fate determinant during transition towards cell differentiation. Elife. 2015;53: 160. doi:10.1017/CBO9781107415324.004
